# C8orf33 dictates DNA double-strand break repair choice by modulating KAT8-mediated H4K16 acetylation

**DOI:** 10.1038/s41419-025-08194-8

**Published:** 2025-11-17

**Authors:** Laila A. Bishara, Enas R. Abu-Zhayia, Marian Nicola, Nabieh Ayoub

**Affiliations:** Department of Biology, Technion - Israel Institute of Technology, Haifa, 3200003 Israel

**Keywords:** Double-strand DNA breaks, Histone post-translational modifications

## Abstract

Homologous recombination (HR) and non-homologous end joining (NHEJ) are two main repair pathways of DNA double-strand breaks (DSBs). The regulation of repair pathway choice is crucial for maintaining genomic stability and preventing carcinogenesis. Consequently, there is increasing interest in elucidating the molecular mechanisms that govern DSB repair pathway selection. Here, we identify chromosome 8 open reading frame 33 (C8orf33) as a novel regulator of DSB repair choice. We show that C8orf33 is a nuclear protein localized predominantly to the nucleolus and is recruited to DSB sites within both nuclear and nucleolar regions. We demonstrate that C8orf33 promotes the recruitment of 53BP1, thus channeling DSB repair toward NHEJ. Consequently, C8orf33 inhibits DNA end resection and counteracts the recruitment of HR factors, BRCA1 and RAD51, to DSB sites. Mechanistically, chromatin profiling analysis reveals that C8orf33 antagonizes the chromatin association of KAT8 acetyltransferase at DSB sites leading to reduced histone 4 lysine 16 acetylation (H4K16ac) levels. Accordingly, the loss of C8orf33 enhances KAT8 chromatin binding that increases H4K16ac levels. This promotes the recruitment of HR factors while suppressing the accumulation of NHEJ factors at DSB sites, thereby favoring HR over NHEJ. Additionally, we demonstrate that the elevated HR activity in C8orf33-deficient cells causes genomic instability, as evidenced by accelerated loss of ribosomal DNA repeats and increases cell death. Collectively, our findings establish C8orf33 as a critical regulator of DSB repair pathway choice, safeguarding genomic integrity.

## Introduction

Eukaryotic cells encounter thousands of endogenous and exogenous DNA lesions on a daily basis that threaten genomic stability. To detect and repair these lesions, cells have evolved intricate and highly coordinated networks known as the DNA damage response (DDR). Among these lesions are DNA double-strand breaks (DSBs), one of the most toxic forms of DNA damage. Improper repair of DSBs has deleterious consequences that can lead to a variety of human diseases such as developmental defects, immunodeficiency, premature aging, neurodegenerative disorders, and cancer [1–5]. The MRE11-RAD50- NBS1 (MRN) complex is responsible for the initial detection of DSB and contributes to ATM activation which phosphorylates Ser139 of the histone variant H2AX (γ-H2AX), the earliest marker of DSBs [6]. Vertebrate cells employ multiple pathways to repair DSBs, each with distinct mechanisms and fidelity [7, 8]. These pathways include non-homologous end joining (NHEJ), homologous recombination (HR), and alternative end-joining (alt-EJ) [2, 6, 9, 10].

HR is initiated by 5′-DNA end resection, which is mediated by the activity of multiple factors including MRE11, CtIP, BRCA1, EXO1 and the heterodimeric DNA2/BLM nuclease-helicase complex, generating long 3′-ssDNA tails of several hundred nucleotides away from the break site [11–16]. The 3′-ssDNA tails are rapidly coated by the heterotrimeric replication protein A (RPA) complex, which protects the exposed DNA from degredation. Subsequently, RPA is displaced with RAD51 protein, forming RAD51-ssDNA nucleoprotein filaments that mediate homology search and strand invasion into a homologous DNA template[11, 17]. NHEJ is initiated by the binding of Ku70-Ku80 heterodimer to DSB ends, which helps protect the DNA ends from excessive processing and degradation. This initial DSB end recognition is supported by 53BP1-RIF1 proteins that recruit the downstream shieldin complex, which acts downstream of 53BP1 to inhibit DNA end resection and promote NHEJ [8, 15, 18–23]. When HR or NHEJ is compromised, cells may employ Alt-EJ, also known as microhomology-mediated end joining (MMEJ), a highly error-prone backup repair pathway [7, 24].

The choice between the different DSB repair pathways is determined by chromatin post-translational modifications (PTMs) at DSB sites, cell cycle phase and the abundance of the various DSB factors [25–31]. For example, BRCA1 competes with 53BP1 recruitment and initiates 5’-DNA end resection, channeling DSB repair towards HR [7, 20, 32–37]. One chromatin PTM that regulates DSB repair choice is Histone 4 lysine 16 acetylation (H4K16ac), which is predominantly catalyzed by K(lysine) acetyltransferase 8 (KAT8), and can also be mediated by K(lysine) acetyltransferase 5 (KAT5), also known as TIP60. While H4K16ac blocks 53BP1, it promotes the recruitment of BRCA1 to DNA damage sites, thus favoring HR repair of DSBs [38–45]. Notably, the balance between HR and NHEJ is crucial to ensure genome stability. For instance, excessive HR at the nucleolar region, evidenced by increased recruitment of HR factor, causes massive ribosomal DNA (rDNA) repeats loss, which is accompanied with the accumulation of mutations and increased cell lethality [46–53].

In this study, we uncover a previously unknown function of C8orf33 in regulating DSB repair pathway choice. We demonstrate that C8orf33 is rapidly recruited to DSBs at the nucleus and the nucleolus. Furthermore, we reveal that C8orf33 suppresses HR and promotes NHEJ. Mechanistically, C8orf33 antagonizes the association of KAT8 with chromatin at DSB sites, leading to a reduction in H4K16ac levels. This decline in H4K16ac facilitates the recruitment of 53BP1 while inhibiting the accumulation of RAD51, thereby steering the repair process toward NHEJ. Consistent with these findings, loss of C8orf33 results in elevated HR activity, which contributes to genomic instability, as indicated by a reduction in rDNA repeats and increased cell lethality.

## Results

### C8orf33 counteracts HR and promotes NHEJ repair of DSBs

**Fig. 1:**
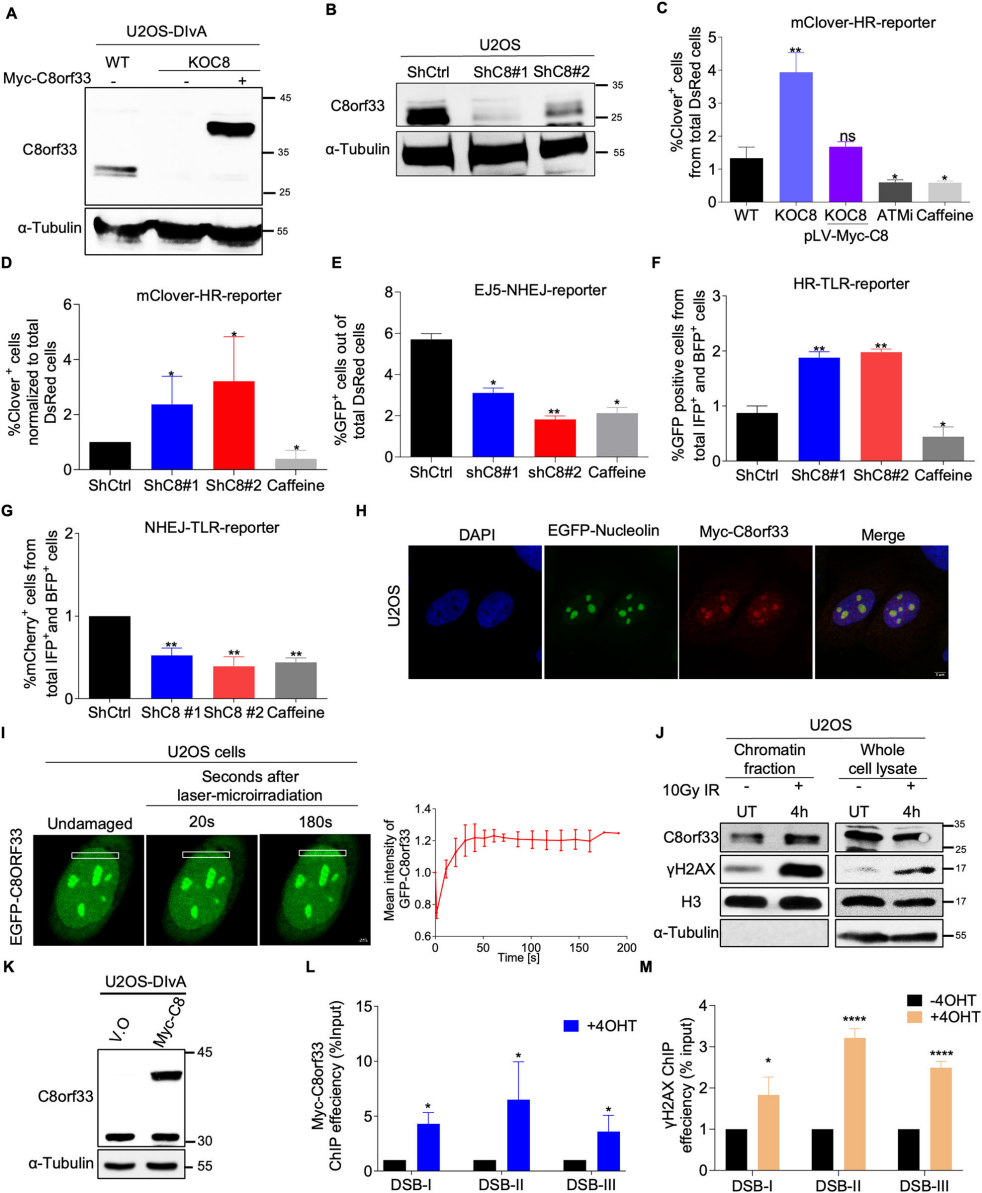
C8orf33 is recruited to DSB sites and regulates DSB repair pathway choice. Western blot shows C8orf33 levels in U2OS-DIvA C8orf33-Knockout (KOC8) cells, and KOC8 cells stably expressing Myc-C8orf33 fusion (Myc-C8) **A** and in U2OS cells expressing scramble shRNA (ShCtrl) and two shRNAs sequences for C8orf33 (ShC8) **B**. **C**, **D** C8orf33 depletion promotes HR of endogenous DSBs induced by Cas9 endonuclease upstream the LMNA gene. Cells were transfected with fluorescence (clover)-based reporter, and HR efficiency was determined at 72 h following transfection, the fluorescence intensity was normalized to the % of cells in S/G2 phases of the cell cycle. **C** U2OS-DIvA WT and KOC8 and dark purple bar is KOC8 expressing Myc-C8. **D** U2OS ShCtrl and ShC8. Error bars represent ±SD from the mean fluorescence intensity of *N* = 3. ATM inhibitor (ATMi, 5 µM) and caffeine (4 mM) were used as positive controls for reduced HR efficiency. **E** C8orf33 depletion reduces NHEJ efficiency. C8orf33-proficient and –deficient U2OS-EJ5 cells containing EJ-5 reporter plasmid stably integrated into their genome were used to determine the efficiency of NHEJ. C8orf33 proficient and deficient U2OS-EJ5 cells were transfected with I*-*Sce-I endonuclease and Red-Monomer (MR) tag. The percentage of GFP-positive cells was analyzed by flow cytometry 48 h post-transfection. 4 mM of Caffeine was added to cells 8 h post transfection and was used as a positive control to detect NHEJ efficiency. Data represent ±SD of *N* = 3. **F**, **G** U2OS-TLR cells show that C8orf33 depletion promotes HR **K** and counteracts NHEJ **L** of DSBs generated by I-Sce-I endonuclease. **F** an increase of ~50% in GFP-positive cells were observed after C8orf33 depletion. 4 mM of Caffeine was used as a positive control. **G** a reduction of ~50–60% in %mCherry positive cells was Observed upon C8orf33 depletion. **H** Immunofluorescence image showing C8orf33 subcellular localization Myc-C8orf33-U2OS cells were transfected with nucleolar nucleolin fused to GFP, stained for Myc (far red) and DNA with DAPI. Scale bar 5 µm **I** C8orf33 recruitment to laser microirradiated sites. Left, representative confocal image showing the relative fluorescence intensity of pEGFP-C8orf33 at damaged sites, cells were exposed to laser microirradiation line indicate recruitment of pEGFP-C8orf33 at 20 s and 180 s post laser induction. Right, the graph shows relative fluorescence (Error bars indicate ±standard deviation (SD) of *N* = 4). Scale bar 2 µm **J** Western blot analysis demonstrating C8orf33 enrichment at the chromatin fraction in untreated (UT) and IR exposed cells following 4 h recovery time. Left, biochemical fractionation. Right, whole cell lysate. Antibodies used, C8orf33, γH2AX a marker of DSB induction, H3 as loading control and tubulin as a control for the chromatin fraction. (representative image of N = 3). **K** Western blot of Myc-C8orf33 overexpression in U2OS-DIvA cells. Antibodies used C8orf33 and tubulin which serves as loading control. ChIP-qPCR analysis for **L** γH2AX and **M** Myc-C8orf33 enrichment upon *AsiSI* induction at 3 DSB sites, DSB-I at the short arm of chromosomes 13, 14, 15, 21 and 22 marking the nucleolar region, DSB-II at chromosome 1 nearby RBMXL1 gene and DSB-III at chromosome 1 nearby CYB651D. The positions of molecular weight markers in all western blots are indicated to the right. Data represent ±SD of *N* = 3. *P*-values of all results were calculated by two-sided Students *t*-test relative to control cells. ns is not significant, ^*^p > 0.05, ^∗∗^*p* < 0.01 and ^***^p > 0.0001.

Previous genome-wide siRNA screen identified C8orf33 as a negative regulator of HR repair of DSBs [54]. Prompted by this, we investigated whether C8orf33 indeed counteracts HR repair. To this end, we measured the efficiency of HR at Cas9-induced DSB within the LMNA gene in both C8orf33 knockout and knockdown cells (Fig. 1A, B, Fig. S1A) [55, 56]. Our findings revealed that C8orf33 depletion leads to a significant increase in HR efficiency (Fig. 1C and D). As positive controls, ATM inhibitor and caffeine were used to counteract HR repair [57, 58]. To further validate the role of C8orf33 in HR regulation, C8orf33 knockout cells were complemented with a vector expressing wild-type C8orf33. Our results demonstrate that expression of C8orf33 restores HR efficiency to levels comparable to those observed in control cells (Fig. 1C). Taken together, these findings suggest that C8orf33 is a negative regulator of HR repair. On the other hand, C8orf33 depletion impairs NHEJ integrity, measured by EJ5-GFP reporter assay [59] (Fig. 1E and S1B). Collectively, these results suggest that C8orf33 protein is a novel regulator of DSB repair choice, facilitating NHEJ and counteracting HR repair of DSBs. To further corroborate this, we simultaneously measured NHEJ and HR efficiency of I-SceI-induced DSBs in cells using traffic light reporter (TLR) [60, 61]. Results show that C8orf33 deficiency increases HR and reduces NHEJ efficiency compared to U2OS-TLR controls (Fig. 1F, G Fig. S1C). Notably, the alterations in HR and NHEJ in C8orf33 deficient cells are not due to changes in cell cycle distribution (Fig. S1D). Altogether, these results suggest that C8orf33 protein is a novel regulator of DSB repair choice, favoring NHEJ and counteracting HR.

### C8orf33 is recruited to DNA DSB sites

To gain insights into how C8orf33 regulates DSB repair, we sought to test whether DNA damage alters its subcellular localization. Interestingly, immunofluorescence (IF) assay revealed that C8orf33 is predominantly a nuclear protein with significant enrichment in the nucleolus (Fig. 1H). Next, U2OS cells expressing EGFP-C8orf33 fusion were subjected to laser microirradiation to induce localized DNA damage within the nucleus of living cells. Time-lapse imaging showed that laser microirradiation leads to a rapid accumulation (within 20 s) of EGFP-C8orf33 at sites of DNA damage. This result demonstrates that EGFP-C8orf33 recruitment is an early event in the DNA damage response (Fig. 1I). To further validate these findings, we examined whether endogenous C8orf33 exhibits similar recruitment to DNA damage sites. U2OS cells were exposed to ionizing radiation (IR) and subjected to biochemical fractionation. Our results revealed that C8orf33 protein is enriched at the chromatin after IR exposure, which primarily generates DSBs. Importantly, the accumulation of C8orf33 at the chromatin fraction is not due to an increase in overall protein levels of C8orf33 after IR treatment (Fig. 1J). Next, we tested C8orf33 recruitment specifically to DSB sites. For this purpose, we utilized genetically engineered U2OS cells stably expressing the cytoplasmic *Asi*SI restriction enzyme fused to the estrogen receptor (ER) hormone-binding domain, referred to as “U2OS-DIvA” cells. Treatment of U2OS-DIvA cells with 4-hydroxy tamoxifen (4OHT) induces the nuclear entry of the *Asi*SI-ER fusion protein, leading to the generation of DSBs at annotated sites across the human genome [62]. Untreated and 4OHT-treated U2OS-DIvA cells stably expressing Myc-C8orf33 fusion were subjected to chromatin immunoprecipitation (ChIP) followed by real-time quantitative PCR (ChIP-qPCR) (Fig. 1K). Results demonstrated that C8orf33 accumulates at three *Asi*SI-induced DSB sites located in both nuclear and nucleolar compartments, which correlates with a significant increase in γH2AX levels (Fig. 1L, M). Collectively, these findings demonstrate that C8orf33 is recruited to DSB sites, suggesting that it has a direct role in regulating DSB repair choice.

### C8orf33 promotes 53BP1 recruitment to DSB sites

**Fig. 2:**
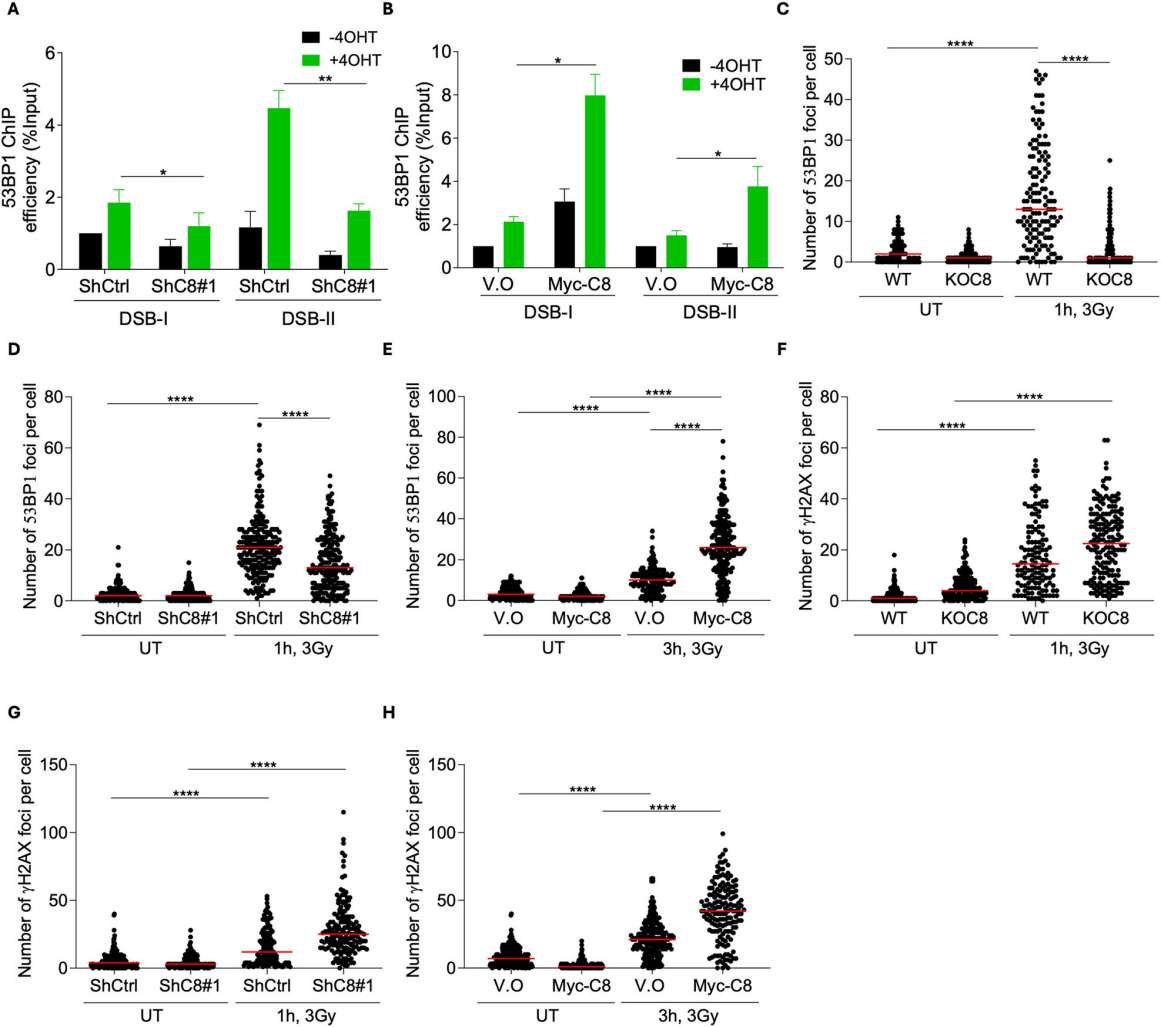
C8orf33 facilitates the recruitment of 53BP1 to DSB sites. **A** Recruitment of 53BP1 to *Asi*SI-induced DSB sites in C8orf33 proficient and deficient cells. ChIP-qPCR for 53BP1 in 4OHT-treated and 4OHT-untreated DIvA cells expressing ShCtrl or ShC8. Error bars represent ±SD of *N* = 3. *P* value was determined two-sided Students *t*-test relative to control cells. **B** Recruitment of 53BP1 to *Asi*SI-induced DSB sites in U2OS cell overexpressing C8orf33. ChIP-qPCR for 53BP1 in 4OHT-treated and 4OHT-untreated DIvA cells expressing Myc-C8 and V.O. Error bars represent ±SD of *N* = 3. *P* value was determined two-sided Students *t*-test relative to control cells. Recruitment of 53BP1 upon IR induction (3 Gy) and recovery for 1 h in **C** U2OS WT and KOC8 cells **D** U2OS ShCtrl and ShC8 cells. Horizontal bars represent mean value of number of 53BP1 foci per cell ± SEM for *N* > 150. *P* value was determined by two-tailed Mann-Whitney test. **E** Recruitment of 53BP1 upon IR induction (3 Gy, 3 h recovery) in U2OS cells expressing either Myc-C8orf33 fusion (Myc-C8) or an empty vector (V.O). Horizontal bars represent mean value of number of 53BP1 foci per cell ± SEM for N > 100. *P* value was determined by two-tailed Mann-Whitney test. Number of γH2AX per cell upon IR induction (3 Gy) and recovery for 1 h in **F** U2OS WT and KOC8 cells, **G** U2OS ShCtrl and ShC8 cells. Horizontal bars represent mean value of number of γH2AX foci per cell ± SEM for *N* > 150. *P* value was determined by two-tailed Mann-Whitney test. **H** Number of γH2AX foci per cells upon IR induction (3 Gy, 3 h recovery). U2OS expressing Myc-C8 and V.O. Horizontal bars represent mean value of number of 53BP1 foci per cell ± SEM for *N* > 100. *P* value was determined by two-tailed Mann-Whitney test. ns is not significant, ^*^p > 0.05, ^∗∗^*p* < 0.01 and ^***^p > 0.0001. ns is not significant, ^*^p > 0.05, ^∗∗^*p* < 0.01 and ^***^p > 0.0001.

Since C8orf33 loss leads to a decrease in NHEJ of DSBs, we posited that C8orf33 promotes the recruitment of the upstream NHEJ factor, 53BP1. To address this, we assessed 53BP1 enrichment at *Asi*SI-induced DSBs. Results show that while C8orf33 depletion reduces the enrichment of 53BP1 at DSB sites, C8orf33 overexpression increases 53BP1 enrichment at DSBs, as evidenced by ChIP-qPCR analysis (Fig. 2A, B). Similarly, C8orf33 depletion impairs 53BP1 IR-induced foci (IRIF) formation and its overexpression increases 53BP1 IRIF formation (Fig. 2C–E and Fig. S2). Notably, the decrease in 53BP1 foci following C8orf33 depletion is not due to changes in 53BP1 global protein levels (Fig. S2A, B). These obseravtions demonstrate that C8orf33 fosters NHEJ by promoting 53BP1 recruitment to DSB sites. Intriguingly, our results show that C8orf33 depletion leads to an increase in γH2AX foci at 1 hour post-IR induction. Similarly, C8orf33 overexpression increases γH2AX foci (Fig. 2F–H and Fig. S2). These findings suggest that precise regulation of C8orf33 expression is critical for proper DSB repair pathway selection.

### C8orf33 inhibits DNA end-resection and the recruitment of HR factors to DSB sites

**Fig. 3:**
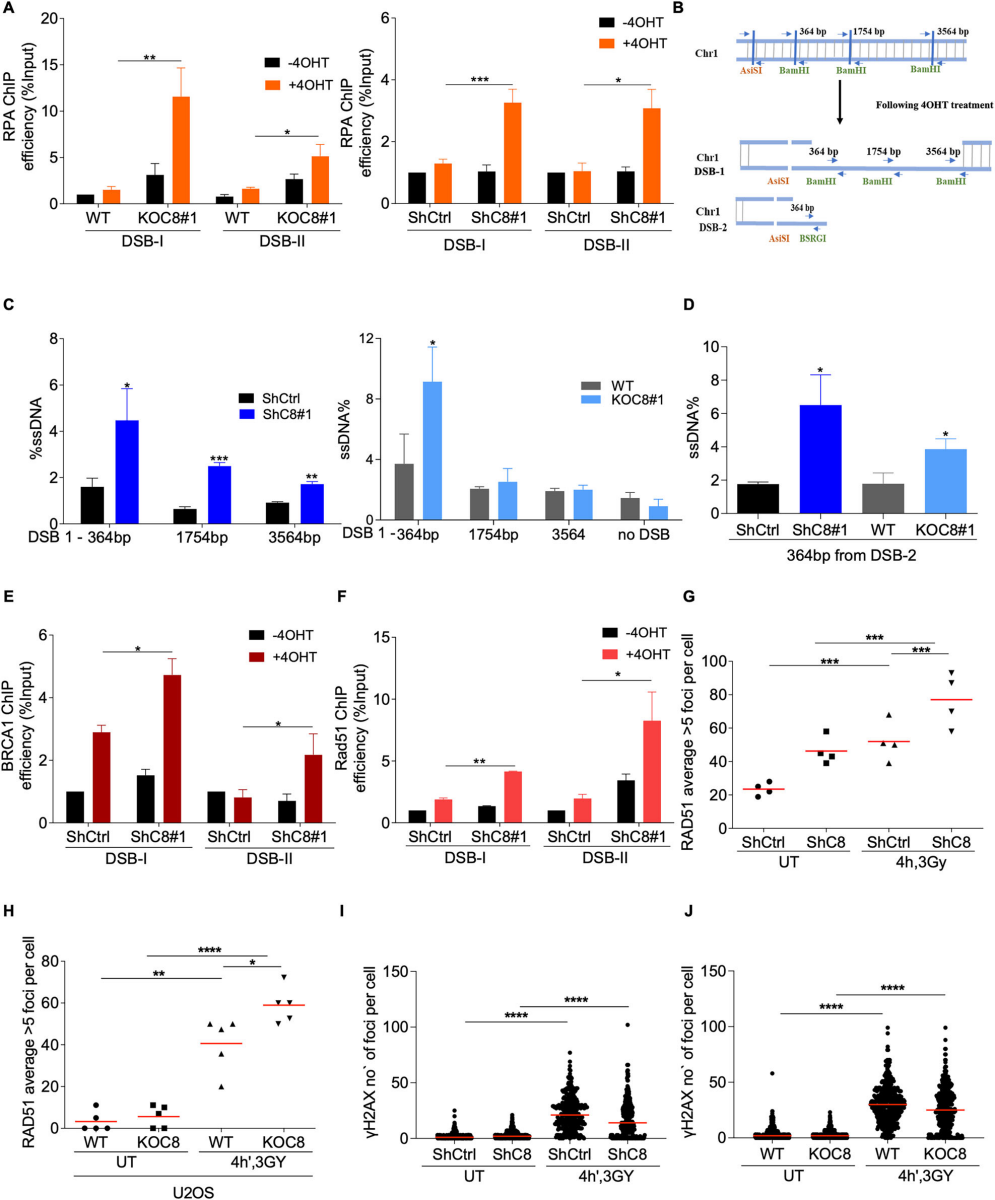
C8orf33 counteracts DNA end resection and HR factors recruitment to DSB sites. **A** RPA enrichment at two *Asi*SI-induced DSB sites. ChIP-qPCR for RPA2 in 4OHT-treated and 4OHT-untreated DIvA cells (left) WT and KOC8 (right) ShCtrl or ShC8. Error bars represent ±SD of *N* = 3. *P* value was determined two-sided Students *t*-test relative to control cells. **B** Schematic representative of DNA end resection assay measuring the extent of ssDNA at two different *Asi*SI-induced DSB sites targeting chromosome 1. Cells were treated with 4OHT, genomic DNA was extracted, then DNA was digested with restriction enzymes nearby *Asi*SI cut sites to distinguish between dsDNA and ssDNA. DSB-1 (digested with BamHI) and DSB-2 (digested with BsRGI) and then subjected to ssDNA quantification using RT-PCR, using primers surrounding the restriction enzyme cut site as indicated in the blue arrows. **C** DNA end resection assay for DSB-1 in DIvA cells. (left) WT and KOC8, (right) ShCtrl or ShC8. **D** Same as in C. The assay was conducted surrounding DSB-2. Error bars represent ±SD of N = 3. *P* value was determined two-sided Students *t*-test relative to control cells. Recruitment of BRCA1 and RAD51 to two *Asi*SI-induced DSB sites. ChIP-qPCR for **E** BRCA1 and **F** RAD51 for 4OHT-treated and 4OHT-untreated DIvA cells expressing ShCtrl or ShC8. Error bars represent ±SD of *N* = 3. *P* value was determined two-sided Students *t*-test relative to control cells. **G** Recruitment of RAD51 upon IR induction (3 Gy) and recovery for 4 h in shCtrl or shC8 U2OS cells. Horizontal bars represent mean value of the percentage of cells representing >5 RAD51 foci per field ± SD *N* = 4 fields. *P* value was determined two-sided Students *t*-test rela*t*ive to control cells. **H** as in G. Recruitment of RAD51 upon IR induction (3 Gy) and recovery for 4 h in WT and KOC8 U2OS cells. Horizontal bars represent mean value of the percentage of cells representing >5 RAD51 foci per field ± SD *N* = 4 fields. **I** Induction of γH2AX upon IR (3 Gy) and recovery for 4 h in shCtrl or shC8 U2OS cells. Horizontal bars represent mean value of number of γH2AX foci per cell ± SEM for N > 300. *P* value was determined by two-tailed Mann-Whitney test. **J** as in **I** Induction of γH2AX upon IR (3 Gy) and recovery for 4 h in WT and KOC8 U2OS cells. Horizontal bars represent mean value of number of γH2AX foci per cell ± SEM for N > 300. *P* value was determined by two-tailed Mann-Whitney test. For all p values ns is not significant, ^*^p > 0.05, ^∗∗^*p* < 0.01 and ^***^p > 0.0001.

To shed molecular insights into the role of C8orf33 in counteracting HR repair of DSBs, we tested the effect of C8orf33 on DNA end resection by measuring the enrichment of RPA2 at DSB sites. ChIP-qPCR analysis shows that C8orf33 depletion leads to a striking increase in RPA2 levels at *Asi*SI-induced DSBs (Fig. 3A). To substantiate this finding, we applied a quantitative DNA resection assay that measures the extent of ssDNA at *Asi*SI-induced DSBs (Fig. 3B) [63]. Results showed that C8orf33 deficiency increases the levels of ssDNA nearby *AsiSI-*induced DSBs, which is accompanied by an increase in BRCA1 and RAD51 recruitment to DSBs (Fig. 3C–F). Similarly, C8orf33 deficiency increases RAD51 IRIF formation, with no detectable changes in the total levels of RAD51 protein (Fig. 3G, H and Fig. S2E). Notably, C8orf33 depletion results in a reduced γH2AX signal at 4 h post-IR induction. These data align with previous work showing that increased HR activity results in decreased γH2AX signal (Fig. 2I, J) [64]. Collectively, our results support the notion that C8orf33 suppresses DNA end-resection and HR factors recruitment through targeting 53BP1 to DSB sites.

### C8orf33 regulates KAT8-mediated H4K16ac

**Fig. 4:**
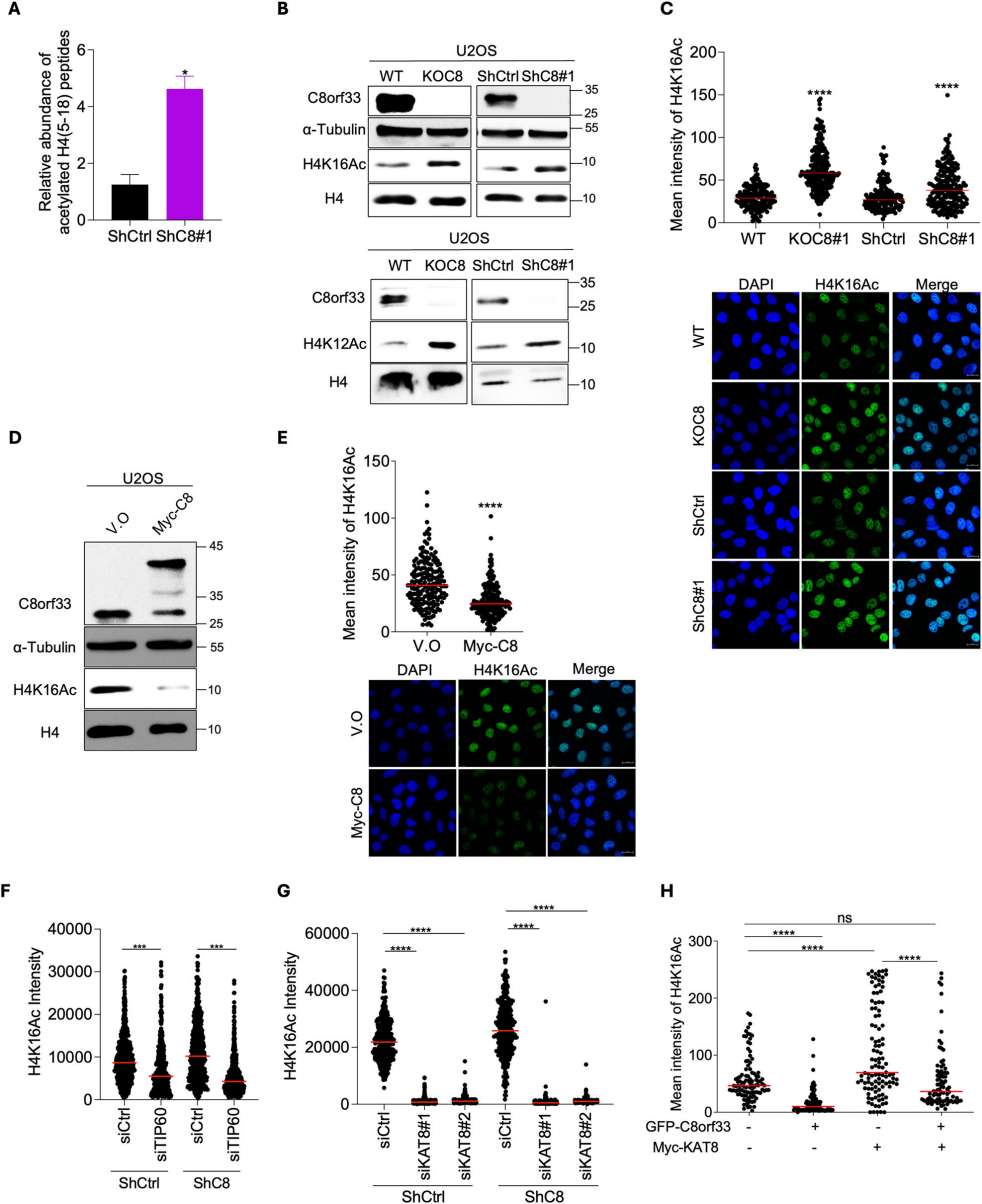
C8orf33 downregulates H4K16Ac levels at DSB sites via counteracting KAT8 chromatin association. **A** Results of quantitative mass spectrometry analysis of H4 [5–18] acetylated peptides in U2OS ShCtrl and ShC8. Acetylated H4 [5–18] peptide intensity were normalized to the intensity of total peptides of H4. Error bars represent ±SD of N = 2. *P* value was determined two-sided Students *t*-test relative to control cells. **B** Western blot for (Top) H4K16ac and (Bottom) H4K12ac levels in C8orf33 proficient and deficient cells. (Left) U2OS WT and KOC8. (Right) U2OS ShCtrl and ShC8. Antibodies used, C8orf33, H4K16Ac, Tubulin, H4 antibodies. Tubulin and H4 were used as loading controls. The positions of molecular weight markers are indicated to the right, representative of *N* = 3. **C** Mean intensity of H4K16ac in C8orf33 deficient cells. Top, quantification of H4K16Ac mean intensity in U2OS KOC8 and ShC8 cells. Bottom, Representative images of H4K16Ac staining (green) in cells, DNA was stained by DAPI. Horizontal bars represent mean value of H4K16ac intensity per cell ± SEM for N > 150. Scale bar 20 µm*. P* value was determined by two-tailed Mann-Whitney test. **D** Western blot for H4K16ac levels in cells overexpressing either V.O or Myc-C8. Antibodies used, C8orf33, H4K16Ac, Tubulin, H4. Tubulin and H4 were used as loading controls. The positions of molecular weight markers are indicated to the right, representative of *N* = 3. **E** Mean intensity of H4K16ac in U2OS cells overexpressing V.O or Myc-C8 fusion. Top, quantification of H4K16Ac mean intensity in V.O and Myc-C8. Bottom, Representative images of H4K16ac staining (green) in U2OS cells chromatin pre-extraction, DNA was stained by DAPI. Horizontal bars represent mean value of H4K16ac intensity per cell ± SEM for *N* > 150. *P* value was determined by two-tailed Mann-Whitney test. Mean intensity of H4K16ac in **F** C8orf33 deficient and C8orf33 and TIP60 mutually deficient cells, **G** C8orf33 deficient and C8orf33 and KAT8 mutually deficient cells. Horizontal bars represent mean value of H4K16ac intensity per cell ± SEM for *N* > 200. **H** Mean intensity of H4K16ac per cell in U2OS cells expressing pEGFP-C8orf33, Myc-KAT8 or co-overexpression pEGFP-C8orf33 and Myc-KAT8. Myc was stained with red, H4K16ac was stained with far-red, and DNA was stained with DAPI. (Left) representative images of un-transfected cells, pEGFP-C8, Myc-KAT8 and cell co-expressing GFP-C8orf33 and Myc-KAT8. (Right) quantification of H4K16ac intensity per cell. Horizontal bars represent mean value of H4K16ac intensity per cell ± SEM for N > 85. Scale bar 10 µm. *P* value was determined by two-tailed Mann-Whitney test. For all *p* values ns is not significant, ^*^p > 0.05, ^∗∗^*p* < 0.01 and ^***^p > 0.0001.

Since chromatin context plays a critical role in DSB repair pathway choice, we sought to test the effect of C8orf33 depletion on histone PTMs. Toward this end, U2OS-proficient and deficient C8orf33 cells were subject to histone acidic extraction followed by liquid chromatography-tandem mass-spectrometry (LC-MS/MS). Strikingly, C8orf33 depletion led to a three-fold increase in H4 (5–18) peptides containing H4K8ac, H4K12ac and H4K16ac (Fig. 4A and S3A). Consistently, western blot (WB) analysis showed similar increase in the levels of H4K12ac and H4K16ac in C8orf33 deficient cells (Fig. 4B). Since H4K16ac is known to regulate DSB repair choice, we sought to further investigate C8orf33 effect on H4K16ac levels. As shown in Fig. 4B, C and S3B-C, C8orf33 depletion increases the levels of H4K16ac both in U2OS and RPE1-hTert^p53-/-^ (RPE1) cells as observed by WB and IF analysis. Furthermore, C8orf33 overexpression leads to a global decrease in H4K16ac levels (Fig. 4D, E and S3D-E). Altogether, we propose that C8orf33 downregulates H4K16ac levels. Next, we sought to address whether the increase in H4K16ac is accompanied by decrease in H4K20me2, thereby limiting 53BP1 recruitment to DSB site. Toward this end, we measured the levels of H4K20me2 levels following C8orf33 depletion. Our proteomics data revealed that C8orf33 depletion increases H4K20me2 levels (Fig. S3G). Therefore, we concluded that defective recruitment of 53BP1 to DSBs in C8orf33-depleted cells is not due to a reduction in H4K20me2, but rather is due to increased H4K16ac levels, which subsequently disrupts 53BP1 binding to H4K20me2. This aligns with previous studies showing H4K20me2 abundance alone is insufficient for 53BP1 recruitment [65, 66]. To further examine how C8orf33 regulates H4K16ac levels, we depleted either KAT8 or TIP60 in both C8orf33 proficient and deficient cells to assess H4K16ac. Our results show reduced H4K16ac levels in TIP60-depleted cells, and cells co-depleted for C8orf33 and TIP60 (Fig. 4F and S4A-B). However, a more pronounce decrease was observed in both KAT8-depleted cells and cells co-depleted for C8orf33 and KAT8 (Fig. 4G and Fig. S4C, D). This result aligns with literature indicating that KAT8 is the primary acetyltransferase for H4K16 [43, 44, 67]. Based on this, we focused on studying the role of C8orf33 in regulating KAT8-mediated H4K16ac. Accordingly, we overexpressed C8orf33, KAT8, or both proteins simultaneously and assessed the levels of H4K16ac at the chromatin fraction. Results showed that while C8orf33 overexpression decreases H4K16ac levels and KAT8 overexpression increases H4K16ac, co-overexpression of C8orf33 and KAT8 restored the levels of H4K16ac comparable to those observed in control cells (Fig. 4H, Fig. S4E). Collectively, our data suggest that C8orf33 influences DSB repair choice, at least in part, via regulating KAT8-mediated H4K16ac levels.

### C8orf33 counteracts KAT8 chromatin association at DSB sites

**Fig. 5:**
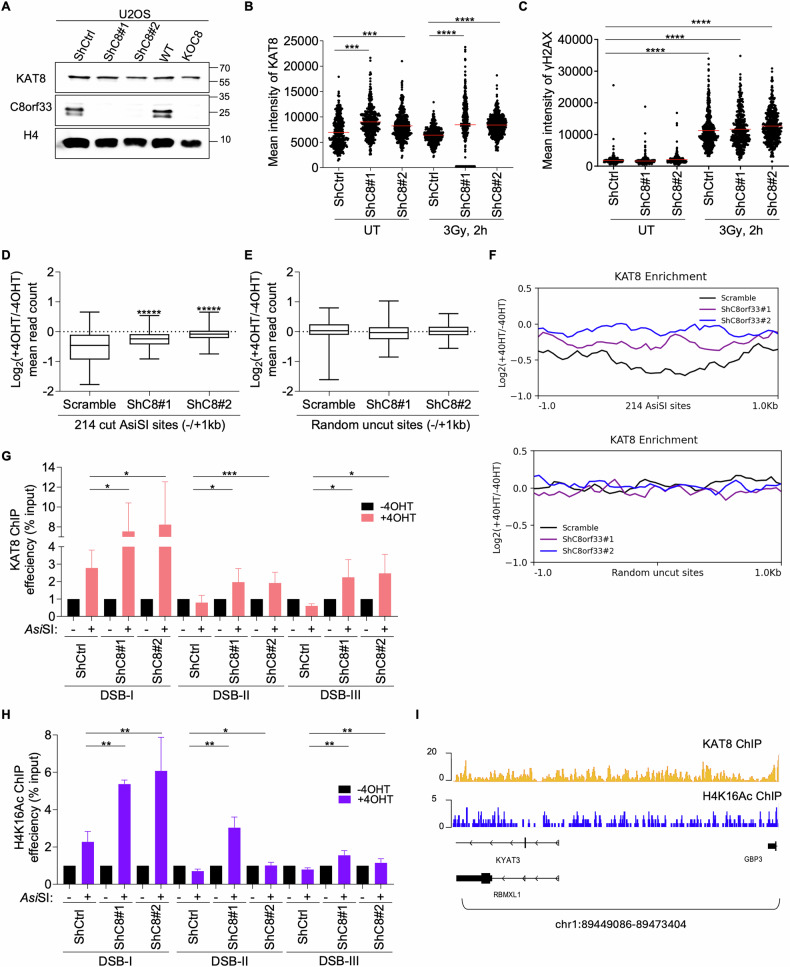
C8orf33 counteracts the chromatin enrichment of KAT8 acetyltransferase at DSB sites. **A** Western blot analysis for KAT8 expression in C8orf33 proficient and deficient cells. Antibodies used, C8orf33, KAT8 and H4 which is used as loading control. The positions of molecular weight markers are indicated to the right, representative of *N* = 3. **B** Quantification of KAT8 mean intensity per cell in U2OS ShCtrl and ShC8 cells. Horizontal bars represent mean value of KAT8 ± SEM for N > 350. *P* value was determined by two-tailed Mann-Whitney test. **C** Quantification of γH2AX mean intensity per cell in U2OS ShCtrl and ShC8 cells. Horizontal bars represent mean value of γH2AX ± SEM for N > 350. *P* value was determined by two-tailed Mann-Whitney test. Boxplot representing the Log2 ratio of KAT8 between treated and untreated DIvA cells expressing either ShCtrl or ShC8 in 1-kb window **D** surrounding the 214 cleaved *Asi*SI sites **E** or surrounding random uncut *Asi*SI sites **E**. The box ends represent the first and third quartiles, the center line represents the median, and the whiskers represent the minimum and maximum values. *P* value was determined by two-tailed Mann-Whitney test, this data is a representation of *N* = 3 independent biological repeats. **F** Distribution of KAT8 log2 ratio between 4OHT-treated and 4OHT-untreated DIvA cells at 1 Kb window surrounding the 214 cleaved *Asi*SI sites in DIvA cells expressing either ShCtrl or ShC8 (top), log2 ratio of KAT8 enrichment surrounding uncut site within the genome (bottom). This data represents 3 independent biological repeats. See also Fig. S5. **G** KAT8 enrichment at 3 different *Asi*SI-induced DSB sites. ChIP-qPCR for KAT8 in 4OHT-treated and 4OHT-untreated DIvA cells either expressing ShCtrl or ShC8. Error bars represent ±SD of N = 3. *P* value was determined two-sided Students *t*-test relative to con*t*rol cells. **H** H4K16ac enrichment at 3 different *Asi*SI-induced DSB sites. ChIP-qPCR for H4K16ac in 4OHT-treated and 4OHT-untreated DIvA cells either expressing ShCtrl or ShC8. Error bars represent ±SD of *N* = 3. *P* value was determined two-sided Students *t*-test relative to con*t*rol cells. For all p values ns is not significant, ^*^p > 0.05, ^∗∗^*p* < 0.01 and ^***^p > 0.0001. **I** Genome browser showing the correlation between KAT8 ChIP-seq data (ShCtrl samples) from the current study and the H4K16ac ChIP-seq data from Clouaire et. Al. 2018.

To gain molecular insights into the mechanism by which C8orf33 regulates KAT8-mediated H4K16ac levels, we first assessed the effect of C8orf33 depletion on KAT8 protein levels. Result showed that C8orf33 depletion does not affect the global levels of KAT8 protein (Fig. 5A). Next, we tested whether C8orf33 depletion affects KAT8 chromatin association during DNA damage. IF analysis revealed that C8orf33 depletion markedly increased chromatin-associated KAT8 levels, both before and after IR (Fig. 5B, C, S5A–E). To confirm these results, we analyzed KAT8 genome-wide binding by ChIP-seq in C8orf33 proficient and deficient U2OS-DIvA cells, both before and after *Asi*SI-induced DSBs. ChIP-seq analysis demonstrated that C8orf33 deficient cells show a prominent increase in KAT8 enrichment at 214 *Asi*SI-induced DSB sites that exhibit the strongest γH2AX signal [62, 68, 69]. In contrast, no detectable changes in KAT8 enrichment were observed near random uncut sites across the genome (Figs. 5D–F and S5F). To validate the ChIP-seq data, we determined the relative enrichment of KAT8 at three *Asi*SI-induced DSB sites. We observed that following 4OHT treatment, C8orf33-deficient cells exhibit a significant increase in KAT8 levels which is accompanied by an increase in H4K16ac levels (Fig. 5G, H), Notably, our data is consistent with previous work showing a strong correlation between KAT8 occupancy and genome-wide H4K16ac enrichment patterns (Fig. 5I) [30]. Collectively, our data provide firm evidence that C8orf33 downregulates H4K16ac during DDR by antagonizing KAT8 chromatin association.

### C8orf33 regulates DSB repair pathway choice via modulating H4K16ac levels

**Fig. 6:**
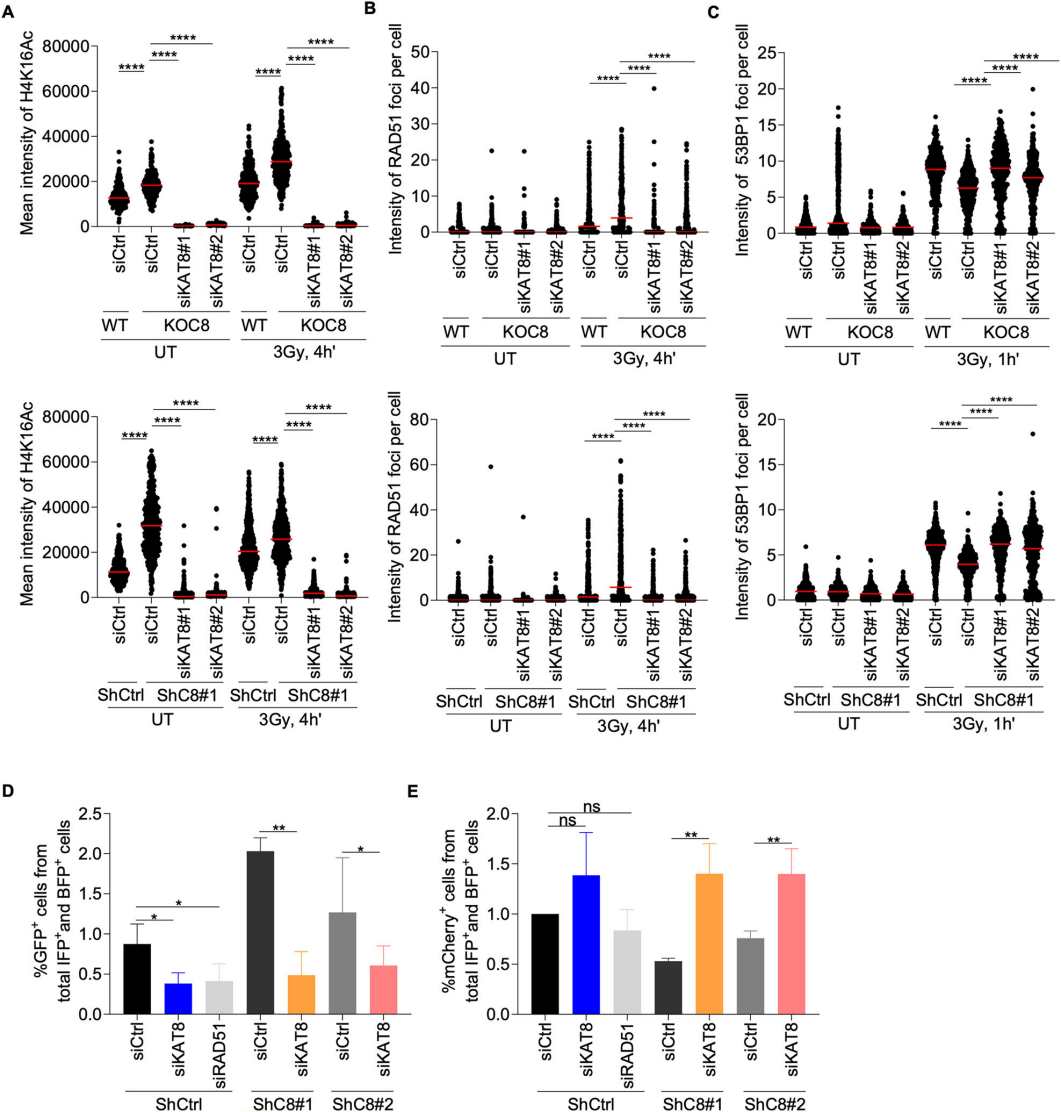
C8orf33-KAT8 axis regulates DSB repair choice via modulating H4K16ac. **A** Mean intensity of H4K16ac in C8orf33 deficient and C8orf33 and KAT8 mutually deficient cells following IR (3 Gy, 4 h recovery). Horizontal bars represent mean value of H4K16ac intensity per cell ± SEM for N > 150. *P* value was determined by two-tailed Mann-Whitney test. **B** Mean intensity of RAD51 foci per cell in C8orf33 deficient and C8orf33 and KAT8 mutually deficient cells following (3 Gy, 4 h recovery). Horizontal bars represent mean value of RAD51 foci per cell ± SEM for N > 150. *P* value was determined by two-tailed Mann-Whitney test. **C** Mean intensity of 53BP1 foci per cell in C8orf33 deficient and C8orf33 and KAT8 mutually deficient cells following IR induction (3 Gy, 1 h recovery). Horizontal bars represent mean value of 53BP1 foci per cell ± SEM for *N* > 200. *P* value was determined by two-tailed Mann-Whitney test. U2OS-TLR cells show that C8orf33 and KAT8 co-depletion counteracts HR **D** and promotes NHEJ **E** of DSBs generated by *I-SceI* endonuclease. **D** A Decrease of ~50% in GFP-positive cells was observed after C8orf33 and KAT8 co-depletion. **E** An increase of ~50%-60% in %mCherry positive cells was observed upon C8orf33 depletion, siRNA for RAD51 was used as a positive control. Error bars represent ±SD of *N* = 3. *P*-values of were calculated by two-sided Students *t*-test relative to control cells. ns is not significant, ^*^p > 0.05, ^∗∗^*p* < 0.01 and ^***^p > 0.0001.

Given that C8orf33 modulates H4K16ac levels, we hypothesized that this epigenetic regulation contributes to its role in determining DSB repair pathway choice. To investigate this, we first examined the impact of C8orf33 and KAT8 co-depletion on the formation of RAD51 and 53BP1 IRIF formation. Strikingly, depletion of C8orf33 alone increases H4K16ac, which was accompanied by increased RAD51 IRIF and decreased 53BP1 IRIF-consistent with enhanced HR and suppressed NHEJ. However, co-depletion of C8orf33 and KAT8 significantly reversed this phenotype, markedly reducing H4K16 Ac levels and RAD51 IRIF while increasing 53BP1 IRIF (Fig. 6A–C, Figs. S6, S7 and S8A). These results demonstrate that the C8orf33-KAT8 axis governs the recruitment of HR (RAD51) and NHEJ (53BP1) factors to DSB sites, thereby dictating repair pathway choice.

To further validate this mechanism, we directly assessed HR and NHEJ efficiency in cells either depleted of C8orf33 alone or co-depleted of C8orf33 and KAT8. Consistent with the IRIF data, C8orf33 deficiency increased HR activity and impaired NHEJ. In contrast, C8orf33-KAT8 co-depletion reversed this bias, restoring NHEJ proficiency at the expense of HR (Fig. 6D, E, Fig. S8B). Collectively, these findings establish that C8orf33 directs DSB repair pathway selection by antagonizing KAT8-mediated H4K16ac deposition, which in turn regulates the balance between HR and NHEJ.

### C8orf33 deficiency drives genomic instability

**Fig. 7:**
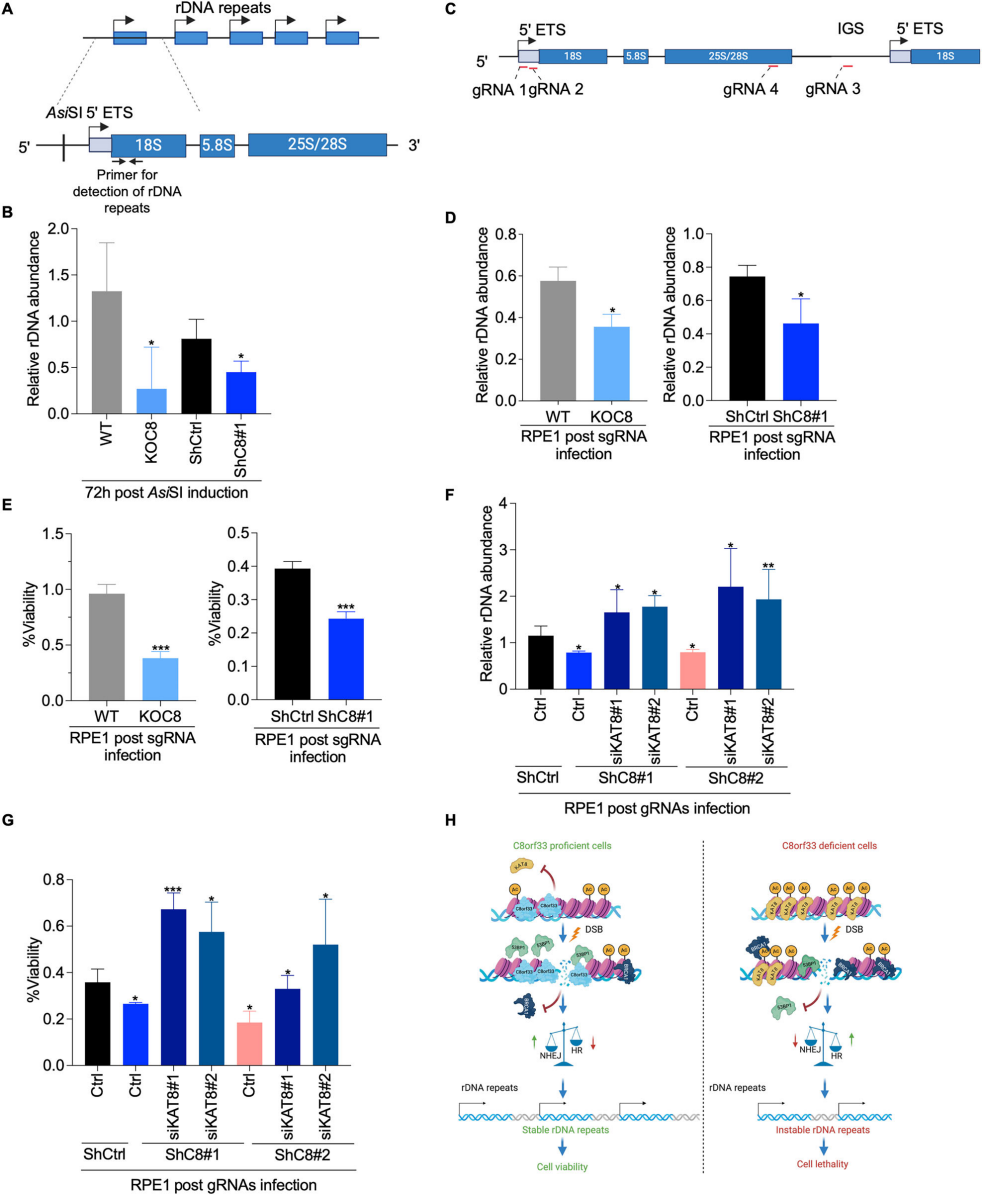
C8orf33 regulates rDNA repeats stability following nucleolar DSBs induction. **A** Schematic representation showing the position of *Asi*SI cut site at the rDNA locus. The primers used to detect rDNA repeats by RT-PCR are indicated. **B** Relative rDNA abundance upon *Asi*SI-induced DSBs. Genomic DNA from U2OS-C8orf33 proficient and deficient cells was extracted and was subjected to RT-PCR surrounding 18S rDNA repeats as indicated in A. Error bars represent ±SD of *N* = 3. *P*-values of were calculated by two-sided Students *t*-test relative to control cells **C** Schematic representation of Cas9 induced targeting 3 nucleolar regions, 5ETS, 28S repeat and the intergenic spacer (IGS). **D** Relative rDNA abundance upon Cas9-induced DSBs. C8orf33 proficient and deficient RPE1 cells were infected with sgRNAs targeting the nucleolus. 72 h post infection Genomic DNA was extracted, and samples were subjected to RT-PCR surrounding 18S repeat. the rDNA abundance is normalized to genomic GAPDH and control samples infected with sgRNA-V.O. Error bars represent ±SD of *N* = 3. *P*-values of were calculated by two-sided Students *t*-test relative to control cells. **E** Short-term growth assay in C8orf33 proficient and deficient RPE1 cells 72 h upon Cas9-induced nucleolar DSBs. Error bars represent ±SD of *N* = 3. *P*-values of were calculated by two-sided Students *t*-test relative to control cells. **F** Same as in **D** RPE1 expressing C8orf33 depletion or co-depletion of C8orf33 and KAT8. Error bars represent ±SD of *N* = 3. **G** Same as in (D) RPE1 expressing C8orf33 depletion or co-depletion of C8orf33 and KAT8. Error bars represent ±SD of *N* = 3. ns is not significant, ^*^p > 0.05, ^∗∗^*p* < 0.01 and ^***^p > 0.0001. **H** A model representing C8orf33 role in DSB repair choice. Right, cells in normal state where C8orf33 is proficient and modulates the levels of KAT8-mediated H4K16ac, upon DSB induction 53BP1 and BRCA1 are efficiently recruited to DSB sites thereby maintaining the balance between HR and NHEJ. C8orf33 limits HR activity which preserves the rDNA repeat stability. Left, C8orf33 deficiency cause KAT8-mediated hyperacetylation thereby blocking 53BP1 recruitment and promotes HR factors recruitment to DSB sites, facilitating HR over NHEJ. This elevated HR activity cause rDNA repeats instability and cell lethality. This graph was created in BioRender. Ayoub, N. (2025) https://BioRender.com/v0fx2wi.

It is well established that excessive homologous recombination (HR) activity leads to loss of ribosomal DNA (rDNA) repeats and subsequent cell lethality [51, 53, 70–72]. Given that C8orf33 deficiency increases HR activity and that C8orf33 localizes prominently in the nucleolus (Figs. 1H and 3A–F), we hypothesized that C8orf33 depletion compromises rDNA repeats stability following nucleolar DSB induction. To test this, we employed two complementary approaches to induce site-specific DSBs within the nucleolar rDNA loci. First, we utilized U2OS-DIvA cells, which harbor *Asi*SI restriction site within the 5′ external transcribed spacer (5′ ETS) of the rDNA repeats (Fig. 7A). C8orf33-proficient and -deficient cells were treated with 4OHT for 4 h and 72 h post treatment genomic DNA was subjected to qPCR to quantify 18S rDNA repeats abundance. Strikingly, C8orf33 depletion resulted in a pronounced reduction in 18S rDNA copy number, suggesting that C8orf33 is essential for maintaining rDNA repeat stability following DSB induction (Fig. 7B).

To further validate these findings, we established an orthogonal system using RPE1 cells stably expressing Cas9 nuclease (Fig. S9A). Cells were transfected with four independent single-guide RNAs (sgRNAs) targeting 5’ETS, 28S and the intergenic spacer of the rDNA locus (Fig. 7C). As shown in Fig. S9B, DSB induction in the nucleolus was confirmed via IF staining for γH2AX. Consistent with our observations in U2OS-DIvA cells, C8orf33-deficient RPE1 cells exhibited a significant reduction in 18S rDNA repeat abundance following nucleolar DSB induction, which is accompanied by increased cellular lethality (Fig. D, E and Fig. S9C, D).

We next sought to elucidate the mechanistic basis of rDNA instability in C8orf33-deficient cells. Given that elevated H4K16ac is associated with increased HR activity, we hypothesized that this modification may contribute to rDNA repeat loss. In support of this, co-depletion of KAT8 and C8orf33 increased rDNA abundance and rescued cellular viability (Fig. 7F, G). These findings suggest that C8orf33 safeguards rDNA stability by modulating HR activity, potentially through regulation of H4K16ac. Collectively, these findings identify C8orf33 as a key guardian of nucleolar genome stability.

## Discussion

Here, we uncovered a novel link between C8orf33 and KAT8-mediated H4K16 acetylation in regulating DSB repair pathway choice (Fig. 7H). Our findings demonstrate that C8orf33 is rapidly recruited to DSB sites and promotes NHEJ while counteracting HR repair of DSBs. This is consistent with a previous genome-wide siRNA screen that identified C8orf33 as a negative regulator of HR repair [54]. We further showed that C8orf33 promotes NHEJ by facilitating the recruitment of 53BP1 while simultaneously inhibiting DNA end resection and the recruitment of BRCA1 and RAD51. These findings are consistent with the established model of 53BP1-BRCA1 competition in dictating DSB repair pathway choice and introduce C8orf33 as a critical upstream regulator of this process [19, 20].

It was previously shown that pre-existing H4K16ac underpins HR repair of DSBs by promoting end resection and the recruitment of HR factors to DSBs [38–42, 73]. In addition to its role in maintaining H4K16ac levels after DSB induction, our data demonstrate that C8orf33 regulates the levels of H4K16ac also before DSB induction, suggesting that C8orf33 influences DSB repair pathway selection prior to DSB formation by monitoring H4K16ac levels.

Mechanistically we show that C8orf33 antagonizes KAT8 chromatin association following DNA damage, thereby reducing H4K16ac levels and shifting the DSB repair balance toward NHEJ over HR. Our results suggest that C8orf33 does not alter KAT8 protein levels but instead restricts its binding to chromatin. A critical question is how C8orf33 affects KAT8 chromatin association. In this regard, previous studies have identified various factors that regulate KAT8 chromatin association, such as PHF20L, which, together with pRB, recruits KAT8 to E2F target genes [74]. Additionally, WDR5 facilitates KAT8 recruitment to chromatin [75]. Future studies should explore whether C8orf33 regulates KAT8 chromatin association through crosstalk with these factors or via an independent mechanism. In addition, Fig. 4F shows that C8orf33 mildly counteracts TIP60-mediated H4K16ac. Future work will be needed to explore the mechanism underlying the crosstalk between C8orf33 and TIP60 activities.

It is well established that histone PTMs play a critical role in regulating DSB repair pathway choice. For example, histone 4 lysine 20 di-methylation (H4K20me2) and histone H2A lysine 15 ubiquitination (H2AK15ub) facilitate 53BP1 recruitment to DSB sites, thereby promoting NHEJ repair [76, 77]. On the other hand, H3K36me3 promotes CtIP recruitment favoring HR repair of DSBs [78]. Additionally, recent work has revealed a crosstalk between H3K36me3 and H4K16ac in coordinating DSB repair choice [79]. Given these findings, future investigations could address how C8orf33 facilitates the crosstalk between H4K16ac and other histone modifications to regulate DSB repair pathway choice.

Although HR is known as an error free process, previous studies showed that HR repair at the nucleolus causes massive alteration in rDNA repeats stability, which is accompanied by elevated micronuclei formation and cell lethality [51, 53, 72]. In line with this, our findings demonstrate that C8orf33 deficiency increases HR causing rDNA repeats loss and increased cellular lethality. These findings support the notion that C8orf33 limits excessive HR at the nucleolus to preserve the genomic integrity of rDNA repeats.

It is well established that DSB repair choice at the nucleolus is influenced by rDNA transcriptional silencing and rDNA relocation to the nucleolar periphery, which are mediated by several factors including Treacle, TOPBP1, RASSF1A and the HUSH complex [46, 72, 80–83]. It remains unknown whether C8orf33 also regulates DSB choice via altering rDNA transcriptional silencing and rDNA relocation to the nucleolar periphery.

Interestingly, several studies showed that C8orf33 is overexpressed in various types of cancers [84–87]. Given the inhibitory effect of C8orf33 on HR repair, we assume that tumors overexpressing C8orf33 are defective in HR repair of DSBs, and consequently exhibit sensitivity to PARP inhibitors (Figs. 1D and 1F, and Fig. 3) [54, 88]. In addition, rDNA repeat instability is found in various human cancers and can be therapeutically targeted using RNA Pol-I inhibitor, CX5641 (currently undergoing phase 1 clinical trials for haematological malignancies) [89–92]. Since C8orf33 loss leads to rDNA instability, we posit that CX5641 inhibitor sensitizes C8orf33-mutated cancers. Future studies should investigate whether C8orf33 expression status can serve as a predictive biomarker to guide therapeutic strategies.

## Material and methods

### Cell culture, drug treatments and transfections

U2OS, HEK293T and RPE-hTert^p53-/-^ cell lines were grown in Dulbeccos modified Eagles medium (DMEM) supplemented with 10% heat-inactivated FBS, 2 mM L-glutamine, 100 units/ml penicillin, and 100 μg/ml penicillin/streptomycin. U2OS-DIvA (AID-*Asi*SI-ER-U2OS) cells were cultured in DMEM medium supplemented with 10% heat-inactivated fetal bovine serum (Gibco), 2 mM L-glutamine (Gibco), 100 unit/mL penicillin, 100 μg/mL streptomycin (Gibco), and 1 mM Sodium Pyruvate in the presence of 400 μg/mL G418. U2OS-DIvA cells infected with lentiviral plasmids expressing either scramble (ShCtrl) or C8orf33-shRNA (ShC8) sequence were cultured with 1 μg/mL puromycin selection. RPE-hTert^p53-/-^ cells infected with pLenti-Cas9-Blast plasmid were cultured with 5 μg/mL Blasticidine. All cell lines were grown in a humidified 37°C incubator with 5% CO2. To induce *Asi*SI-dependent double-strand breaks (DSBs), cells were treated with 300 nM (z)-4-Hydroxytamoxifen (4OHT) for 4 h. Plasmid DNA and siRNA transfections were conducted using Polyethylenimine (PEI) and Lipofectamine RNAiMax, according to the manufacturer’s protocols. The siRNA used to target KAT8 in this study are, siKAT8#1(5-GAGGAAUUCUACAUACGUGG-3), siKAT8#2 (5-CAAAAGCGCAAGCAUGAUGAGAUCA-3) and siTIP60(5-AAGCUUUUCUACGUCCAU-3).

### Generation of C8orf33-knockout cell line using CRISPR/Cas9 methodology

To generate C8orf33 knockout in U2OS, DIvA-U2OS and RPE-hTert^p53-/-^ cells, C8orf33 single guid RNA (sgRNA) was cloned into pSpCas9(BB) − 2A-GFP (PX458) (Addgene #48138) vector and transfected to cells. At 24 h after transfection, GFP-positive cells were sorted using BD LSRFortessa™ cell analyzer (BD Biosciences) and plated in 96-well plates at a dilution of one cell per well. Single clones were screened for C8orf33 knockout using western blot and validated by sequencing. The sequences of C8orf33 sgRNA are included in Table S4.

### Generation of expression vectors

To generate EGFP-C8orf33 expression vector, C8orf33 was amplified from genomic DNA using indicated primers (Table S4) and cloned into pEGFP-C1 expression plasmid. To constitutively express C8orf33, full-length C8orf33 including Myc tag was subcloned to Lenti-Cas9-Blast plasmid followed by lentivirus generation and transduction. To generate sgRNA plasmids targeting the nucleolar regions, sgRNAs sequences (Table S4) were cloned to pLCKO lentiviral vector. Scrambled and C8orf33-targeting short hairpin oligonucleotides were annealed and cloned into the pLKO.1-TRC lentiviral vector that had been digested with EcoRI and AgeI. The resulting lentiviral vectors were verified by DNA sequencing.

### Generation of lentiviral particles and cell Transduction

To generate viral particles, HEK293T cells were co-transfected in a 10 cm dish with the lentiviral plasmid (1.64 pmol) and viral packaging plasmids psPAX2 (1.3 pmol) and pMD2.G (0.72 pmol). 48 h post transfection, the media containing the viral particles was collected and filtered using 0.45 μm filters. The viral particles were then used to infect U2OS, U2OS-DIvA and RPE-hTert ^p53-/-^ cells. Cells infected with shRNA-expressing plasmids were maintained in 1 μg/ml Puromycin for one week, while those infected with pLenti-Myc-C8orf33-Blast or Lenti-Cas9-Blast were cultured in 5 μg/ml Blasticidin for one week.

### Western blot

Protein extracts were prepared using Hot-lysis buffer (1% SDS, 5 mM EDTA, 50 mM Tris, pH 7.5) as previously described [93]. The antibodies used for Western blotting and their dilutions are detailed in Table S1. Histone H3, Histone H4, and Tubulin served as loading controls, while γH2AX was employed as a marker of DNA damage induction. Protein samples presented in separate panels were analysed in different gels, unless otherwise noted in the figure legends. Molecular weight sizes of the proteins were indicated on the left side of the Western blots. The secondary antibodies used in this assay are: anti-rabbit IgG-HRP (Jackson ImmunoResearch cat.no. 111–035-003, 1:20,000), anti-mouse IgG-HRP (Amersham cat.no. NXA931, 1:10,000).

### Biochemical fractionation

Biochemical fractionation was performed following the methods described by [68]. Briefly, U2OS cells were either untreated or exposed to 10 Gy IR, followed by recovery period of 4 h. Cells were lysed in Buffer A for five minutes at 4°C. After centrifuging at 1500× g for five minutes at 4°C, the supernatant was discarded. The pellet was then resuspended in Buffer B (3 mM EDTA, 0.2 mM EGTA, 1 mM DTT, PMSF, and a protease inhibitor mixture) and incubated on ice for 10 min before another centrifugation at 1700× g for 5 min at 4°C. To prepare the chromatin-bound fraction, the pellet was resuspended in Hot-lysis buffer (1% SDS, 5 mM EDTA, 50 mM Tris, pH 7.5), boiled for 15 min, and sonicated with two 15-s pulses at 38% amplitude. Following centrifugation at maximum speed for 20 min at 12°C, the supernatant was collected. Chromatin-bound fractions were subjected to Western blot analysis and immunoblotted with the specified antibodies.

### Laser micro-irradiationn

Cells were subjected to laser microirradiation as previously described [61]. In brief, cells were plated on Ibidi flourodishes (Cat#81158) and pre-sensitized with 1 μg/μl Hoechst 33342 dye for 10 min at 37°C. Laser microirradiation was performed using LSM-700 inverted confocal microscope equipped with a CO2 module and a 37°C heating chamber. DNA damage was induced by micro-irradiating a specific region of the nucleus with 15 iterations of a 405 nm laser beam. Time-lapse images were captured using a 488 nm laser, and signal intensity at the damaged sites was measured using Zen 2009 software.

### DNA and RNA extraction, reverse transcription and quantitative real-time PCR

DNA was isolated using Macherey-Nagel Nucleospin kit (2406-002) and RNA was isolated from cells using the TRIzol reagent according to the manufacturer’s instructions (Ambion). 1 μg RNA was used for cDNA synthesis using qScript cDNA Synthesis Kit (Quanta) with random primers. DNA and mRNA levels were measured using Step-One-Plus real-time PCR System (Applied Biosystems) using the indicated primers (Table S4) and the Fast SYBR Green Master mix (Applied Biosystems). Data analysis and quantification were performed using StepOne software V2.2 supplied by Applied Biosystems. GAPDH gene was used as a housekeeping gene.

### Endogenous homologous recombination assay

The homologous recombination repair assay was performed using Cas9-mediated knock-in of the green fluorescent protein, mClover, nearby the first exon of the LMNA gene, as previously described [55]. Briefly, cells were plated in 6-well plates and co-transfected with 1.6 μg of the pX330-LMNA-gRNA1 plasmid, which expresses Cas9 and gRNA targeting exon 1 of the LMNA gene, along with 0.4 μg of the pCR2.1-CloverLamin plasmid containing the HR donor sequence. An additional 0.4 μg of pDsRed-Monomer-C1 was included for transfection control, unless specified otherwise in the figure legend. 12–16 h post transfection media was changed, and ATMi (5 μM) or caffeine (4 mM) was added where indicated. 72 h after transfection, cells were collected and analysed by flow cytometry. HR efficiency was calculated as the percentage of mClover-expressing cells among DsRed-Monomer positive cells. Data are presented as mean ± standard deviation from three independent experiments.

### NHEJ-reporter assay (EJ5)

EJ-5 reporter assay was performed as previously described [59]. Briefly, U2OS-EJ5 cells containing EJ-5 reporter plasmid stably integrated into their genome were used to determine the efficiency of NHEJ. U2OS-EJ5 were plated in 6 well plate and co-transfected with 1 μg IsceI endonuclease and 0.2 μg pDsRed-Monomer-C1 for transfection control. 12 − 16 h post transfection, culture medium was renewed and where indicated, Caffeine (4 mM) was added. 48 h post-transfection, cells were collected and analyzed by flow cytometry. NHEJ efficiency is the percentage of DsRed-Monomer positive cells that express GFP reporter gene. Data are presented as mean ± standard deviation from three independent experiments.

### Traffic light reporter (TLR) assay

TLR assay was performed as previously described (Certo et al. [60]; Schmidt et al., 2015; Abu-Zhayia et al., 2017). In brief, U2OS-TLR cells stably expressing either scramble or shRNA sequences targeting C8orf33 were co-transfected with plasmids expressing I-SceI nuclease fused to infrared fluorescent protein (IFP) and donor plasmid expressing GFP donor sequence fused to blue fluorescent protein (BFP). 48 h post transfection, cells were harvested, and GFP and mCherry signals (reflecting HDR and NHEJ, respectively) were measured by Cytek Aurora©. The signal of mCherry or GFP were measure out of IFP and BFP double positive cells. Data are presented as mean ± standard deviation from three independent experiments. Results of shC8 cells were normalized to shCtrl cells. For experiments with KAT8 siRNA, cells were transfected with siRNA targeting KAT8 gene 16 h prior to IsceI transfection.

### Cell cycle analysis by flow cytometry

Flow cytometric analysis was conducted as previously described [61]. In brief, cells were fixed with ice-cold 75% ethanol, and DNA was stained with 100 mg/ml propidium iodide (Sigma-Aldrich) in phosphate buffer solution (PBS) containing 0.5 mg/ml DNase-free RNase A (Sigma-Aldrich) and 0.1% Triton-X-100. Samples were analysed using a BD LSR-II flow cytometer (Becton Dickinson), and the data were analyzed using FCS Express software. HR efficiency was normalized to the % of cells in S/G2 phases.

### Immunofluorescence assay

Cells were seeded onto glass coverslips 24 h prior to IR induction as indicated in the figure legends. Cells were fixed with 4% (wt/vol) paraformaldehyde (PFA) for 10 min, permeabilized with washing buffer (0.15% Tween 20 and 0.15% Triton X-100 in PBS), blocked with blocking buffer (4% (wt/vol) BSA, 0.15% Tween 20 and 0.15% Triton X-100 in PBS) for 1 h at room temperature and incubated with the indicated antibodies for 1 h at 37 °C. Excess antibody was washed three times with washing buffer and cells were stained with secondary antibodies for 1 h at room temperature in the dark, and then washed as above. Coverslips were mounted onto glass slides using VECTASHIELD® Antifade Mounting Medium with DAPI (Vectorlabs). For immunofluorescence staining of γH2AX, 53BP1, RAD51, H4K16ac and KAT8 cells were pre-extracted with 0.25% Triton X-100 in PBS for 7 min on ice prior to fixation. Images were acquired using inverted Zeiss LSM-700 confocal microscope. Image analysis was performed using ImageJ software. The primary antibodies used for immunofluorescence (IF) are included in supplementary Table S1. The secondary antibodies used for IF anti-rabbit IgG Alexa Fluor®488 (Invitrogen cat.no. A-21206, 1:500), anti-mouse IgG Alexa Fluor®488 (Invitrogen cat.no. A-21202, 1:500), anti-rabbit Alexa Fluor®568 (Invitrogen cat.no. A10042, 1:500), anti-mouse Alexa Fluor®568 (Invitrogen cat.no. A10037, 1:500), anti-rabbit Alexa Fluor®647 (Invitrogen cat.no. A-31573, 1:500), anti-mouse IgG Alexa Fluor®647 (Invitrogen cat.no. A32787, 1:500).

### In gel proteolysis and mass spectrometry analysis

The protein samples were brought to 8.5 M Urea, 100 mM ammonium bicarbonate and 10 mM DTT. Protein amount was estimated using Bradford readings. The samples were reduced (60°C for 30 min), modified with 35.2 mM iodoacetamide in 100 mM ammonium bicarbonate (room temperature for 30 min in the dark) and digested in 1.5 M Urea, 17.6 mM ammonium bicarbonate with Arg-C (Promega), overnight at 37oC in a 1:50 (M/M) enzyme-to-substrate ratio. The resulted peptides were desalted using Oasis HLB 96-well µElution Plate (Waters)/homemade C18 stage tip, dried and re-suspended in 0.1% Formic acid in 2% acetonitrile. The resulting peptides were analyzed by LC-MS/MS using an Exploris 480 mass spectrometer (Thermo) fitted with a capillary HPLC (Evosep One). The peptides were loaded onto a 15 cm, ID 150 µm, 1.9-micron Performance column EV1137 (Evosep). The peptides were eluted with the built-in Xcalibur 15 SPD (88 min) method. Mass spectrometry was performed in a positive mode using repetitively full MS scan (m/z 250–1200) followed by High energy Collision Dissociation (HCD) of the 20 most dominant ions ( > 1 charges) selected from the full MS scan. A dynamic exclusion list was enabled with exclusion duration of 30 s. The mass spectrometry data was analyzed using Proteome Discoverer 2.4 (Thermo) using Sequest search engine, searching against the human proteome from the Uniprot database with mass tolerance of 20 ppm for the precursor masses and 0.02 Da for the fragment ions. Oxidation on methionine, methylations (mono, di and tri) on lysine, Acetylation on lysine, ubiquitination on lysine, phosphorylation on serine, threonine and tyrosine and protein N-terminus acetylation were accepted as variable modifications and carbamidomethyl on cysteine was accepted as static modifications. Minimal peptide length was set to five amino acids and a maximum of two miscleavages was allowed. The data was quantified by label free analysis using the same software. Peptide- level false discovery rates (FDRs) were filtered to 1% using the target-decoy strategy. Acetylated H4[5–18] peptide intensity in each sample was normalized to total H4 intensity and presented as mean Acetylated H4 [5–18] peptide abundance relative to control sample from 2 biological repeats. The mass spectrometry proteomics data have been deposited to the ProteomeXchange Consortium via the PRIDE (PubMed ID: 39494541) partner repository with the dataset identifier PXD062780.

### Chromatin immunoprecipitation followed by real-time quantitative PCR (ChIP-qPCR)

ChIP assays were conducted as previously described [94]. Briefly, cells were plated in 150 mm dishes. When they reached approximately 80% confluency, the cells were treated with 300 nM 4OHT for 4 h. The cells were fixed with 1% PFA for 10 min at room temperature, and cross-linking was halted with 0.125 M glycine for 5 min. After cell lysis, DNA was sheared to 300–500 bp using a Vibra Cell sonicator (15 sec ON, 30 sec OFF, 38% duty, 20 cycles). 5% of each supernatant was retained as an input control and processed with the cross-linking reversal step. Chromatin was immunoprecipitated with 1 μg of the indicated antibodies and 10 μl of Protein A/G magnetic beads overnight at 4°C. Next the immunoprecipitated complex was reverse cross-linked using 1% SDS overnight at 65°C. Precipitated DNA was purified using the Macherey-Nagel Nucleospin kit. Both input and IP samples were analyzed by qPCR using primers listed in Table S3. ChIP efficiency was normalized to the total percentage of input DNA that was immunoprecipitated. All ChIP-qPCR data are presented as mean and standard deviation from three representative experiments out of three biological repeats.

### Chromatin immunoprecipitation followed by sequencing (ChIP-seq)

For ChIP-seq, cell treatments and chromatin immunoprecipitations were performed as described above. Libraries were constructed simultaneously using NEBNext Ultra II DNA Library Prep Kit for Illumina (NEB, cat no. E7645), according to the manufacturers protocol, without fragmentation. ∼5 ng of purified DNA (average size 300-500 bp) was used for library preparation. Samples were sequenced using Illumina NextSeq (single-end with unique molecular identifiers, 75 bp reads; KAT8) at the Azrieli Technion Genomics Center (ATGC), Technion- Israel institute of technology. Average read depth of 50 million reads per sample was obtained. Quality assessment was performed using FastQC to verify the sequencing data pass the quality standard (http://www.bioinformatics.babraham.ac.uk/projects/fastqc/).

### ChIP-seq data analysis

ChIP-seq read processing was done as previously described [68]. Briefly, samples were aligned to using GRCh38/hg38 human genome assembly bowtie2 [95]. The resulting aligned reads, initially in SAM format, were converted into BAM format and then sorted, indexed, and quality-filtered using SAMtools [96]. For each BAM file, the bamCoverage tool from deepTools was utilized to calculate coverage and normalize each sample based on Reads Per Kilobase per Million mapped reads. Finally, the coverage files for each biological replicate were merged into BigWig format using the “add” option in bigwigCompare (deepTools) for further analysis. As for average ChIP-seq profiles, computeMatrix (deepTools) was used to calculate the ChIP-seq intensity around specific AsiSI and non-AsiSI at each 40 bp bin size. Log2 ratio tracks were created using the bigwigCompare tool from deepTools (http://deeptools.readthedocs.io), average log2 ratios around specific AsiSI and non- AsiSI sites were computed using computeMatrix. The normalized read count profiles and log2 ratio profiles were then visualized using plotProfile (deepTools). The random non- AsiSI and AsiSI sites were obtained from previously published data [68]. Publicly available previously reported high-throughput sequencing data for H4K16ac ChIP-seq in DIvA cells were downloaded from ArrayExpress: E-MTAB-5817 and re-analyzed using the pipeline described in this study [30, 68].

### Quantitative DNA end resection assay

Quantification of the extent of DNA end resection is conducted as previously described [63, 97], with some modifications. U2OS-DIvA cells were plated 24 h prior to 4OHT treatment to induced AsiSI -induced DSBs. Then cells were harvested at a concentration of 6 × 106 cells/ml. A 50 μl cell suspension was placed onto Parafilm to form a solid agar ball, which was then transferred into a 1.5 ml Eppendorf tube. The agar ball underwent treatment with 1 ml of ESP buffer (containing 0.5 M EDTA, 2% N-lauroylsarcosine, 1 mg/ml proteinase-K, 1 mM CaCl2, pH 8.0) for 20 h at 16°C with continuous rotation. Then, the agar balls were treated with 1 ml of HS buffer (comprising 1.85 M NaCl, 0.15 M KCl, 5 mM MgCl2, 2 mM EDTA, 4 mM Tris, 0.5% Triton X-100, pH 7.5) for another 20 h at 16°C with rotation. Afterward, the sample was washed six times with 1 ml of phosphate buffer (containing 8 mM Na2HPO4, 1.5 mM KH2PO4, 133 mM KCl, 0.8 mM MgCl2, pH 7.4) for 1 h each at 4°C, with rotation. The agar was then melted by heating the tube in a 70°C heat block for 10 min. The melted sample was diluted 15-fold with pre-heated 70°C deionized water, combined with an equal volume of suitable 2× NEB restriction enzyme buffer, and stored at 4°C for later use. The extent of DNA resection near specific DSBs was assessed using qPCR. The detailed sequences for the qPCR primers and probes can be found in (Table S4). Genomic DNA samples of approximately 140 ng in 1× NEB restriction enzyme buffer 4 were subjected to digestion or mock digestion with 20 units of BamHI-HF for DSB-1, BsrGI for DSB-2, or HindIII-HF for no-DSB region (New England Biolabs) at 37°C overnight. This digestion allows detection of ssDNA over dsDNA DNA fragments and since DSB-1 is nearby several recognition sites of BamHI, it allow to track the length of DNA end resection from the DSB site. For DSB-1, and no DSB qPCR reaction, around 20 ng of digested sample was used and served as the template in a 25 μl reaction mixture containing 12.5 μl of 2× TaqMan Universal PCR Master Mix (IDT), alongside 0.5 μM of each primer and 0.2 μM of the probe. The assay was conducted in StepOne software V2.2 supplied by Applied Biosystems. As for DSB-2, 20 ng of digested sample was used for PCR reaction and quantification was done using Fast SYBR Green Master mix (Applied Biosystems) and primers surrounding BsRGI cut site. Data analysis and quantification were performed using StepOne software V2.2 supplied by Applied Biosystems. The method described in reference [63] was used to calculate the percentage of single-stranded DNA (ssDNA%) formed by resection at the target sites. For each sample, the △Ct value was determined by subtracting the Ct value of the mock-digested sample from that of the digested sample. The ssDNA% was then calculated using the formula: ssDNA% = 1/(2^(△Ct-1) + 0.5) *100.

### Short-term growth delay assay

Cells stably expressing Cas9 were infected with PLCKO-sgRNA targeting 4 sites within the nucleolar region at a ratio of 1:1:1:1, sgRNA sequences are stated in (Table S4) and PLCKO-V.O as a negative control. 24 h post infection cells were seeded in 96-well plates in triplicates at a density of 4000 cells per well. 72 h post-seeding, cell viability was measured using CellTiter 96® AQueous One Solution Cell Proliferation Assay (Promega) following the manufacturers protocol, and absorbance was measured using Epoch Microplate Spectrophotometer (BioTek). Cell viability was normalized to the viability of cells infected with PLCKO-V.O.

### PCR analysis to detect rDNA repeats

Genomic DNA was extracted using Exgene Cell SV mini (GeneAll, lot. 10623H28069) according to the manufacturer’s description. DNA was isolated, and 10 µg of DNA was used to measure rDNA abundance using the One-Plus real-time PCR System (Applied Biosystems) with the primer targeting 18S rDNA repeat (Table S4) and the Fast SYBR Green Master Mix (Applied Biosystems). Data analysis and quantification were performed using StepOne software V2.2 supplied by Applied Biosystems. The genomic GAPDH primer was used as a housekeeping gene.

### Statistical analysis

The number of replicates in each experiment is indicated in the figure legends and/or methods. Immunoblots were repeated at least 3 times with one representative experiment presented. For microscopy-based analysis, images were obtained randomly from each sample and analysed together with control samples using the same analysis pipeline. Researchers were not blinded during data collection and analysis as the study design included appropriate controls and did not require blinding. For ChIP-qPCR analysis the data is a representative of 3 technical repeats and extracted out of 3 biological replicates. Data collection and analysis of different conditions were performed at the same time and using identical methods. Microscopy and high-throughput acquisition and analysis was performed automatically without bias between different samples. Statistical tests for each experiment are indicated in the figure legend.

## Supplementary information

Supplementary data

Original western blots

## Data Availability

ChIP-sequencing data have been deposited in ArrayExpress and are available through accession number E-MTAB-15055. The mass spectrometry proteomics data have been deposited to the ProteomeXchange Consortium via the PRIDE repository [98] with the dataset identifier PXD062780. Source data of this study are available in Mendeley data with the identifier 10.17632/6bvcmggy99.1. In addition, the uncropped western blots conducted in this study are provided in with this paper. All data and protocols described in this study are available from the corresponding author upon request.
