# Supplementary material for: C8orf33 dictates DNA double-strand break repair choice by modulating KAT8-mediated H4K16 acetylation
Source: Cell Death Dis. 2025 Nov 17;16(1):834. doi: 10.1038/s41419-025-08194-8 (PMC12624059; doi:10.1038/s41419-025-08194-8)
Supplement: Supplementary file 1 — Supplementary data [file 41419_2025_8194_MOESM1_ESM.docx]

***Supplemental information*….**

**C8orf33 dictates DNA double-strand break repair choice by modulating KAT8-mediated H4K16 acetylation**

Laila A. Bishara^1^, Enas R. Abu-Zhayia^1^, Marian Nicola^1^, Nabieh Ayoub^1^*

1. Department of Biology, Technion - Israel Institute of Technology, Haifa 3200003, Israel.

*Corresponding author: Prof. Nabieh Ayoub, Faculty of Biology

Technion - Israel Institute of Technology

Haifa 3200003, Israel.

Tel: +972-4-8294232

Email: ayoubn@technion.ac.il

**This file includes:**

1. Supplementary Figures and Figure legends 1-9
2. Supplementary Tables 1-4.

**
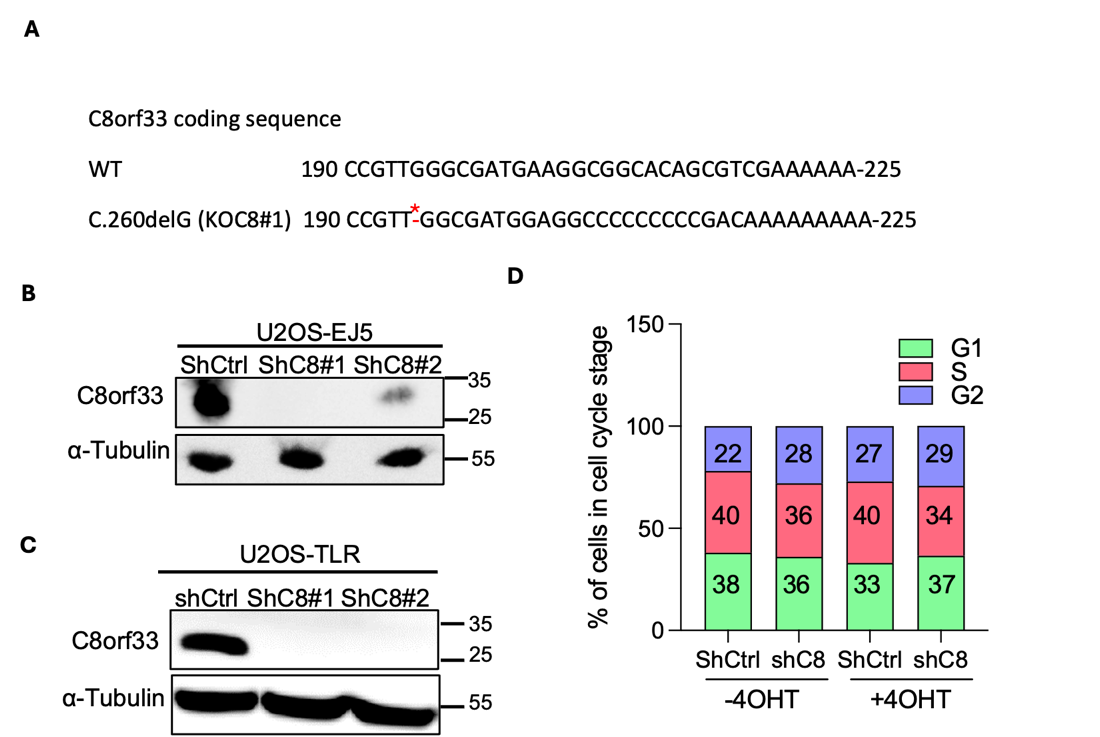
**

**Supplementary Figure 1:** Related to Figure 1. **(A)** DNA sequence alignment of the region in C8orf33 exon 1 containing the position of the 260delG* mutation introduced using CRISPR-Cas9 system (highlighted in red). **(B-C)** Western blot analysis for C8orf33 proficient and deficient cells. (B) U2OS-EJ5-ShCtrl and ShC8. (C) U2OS-TLR-ShCtrl and ShC8. Tubulin was used as loading control. The positions of molecular weight markers are indicated to the right. (**D)** cell cycle analysis for ShCtrl and ShC8 before and after AsiSI induction.

**
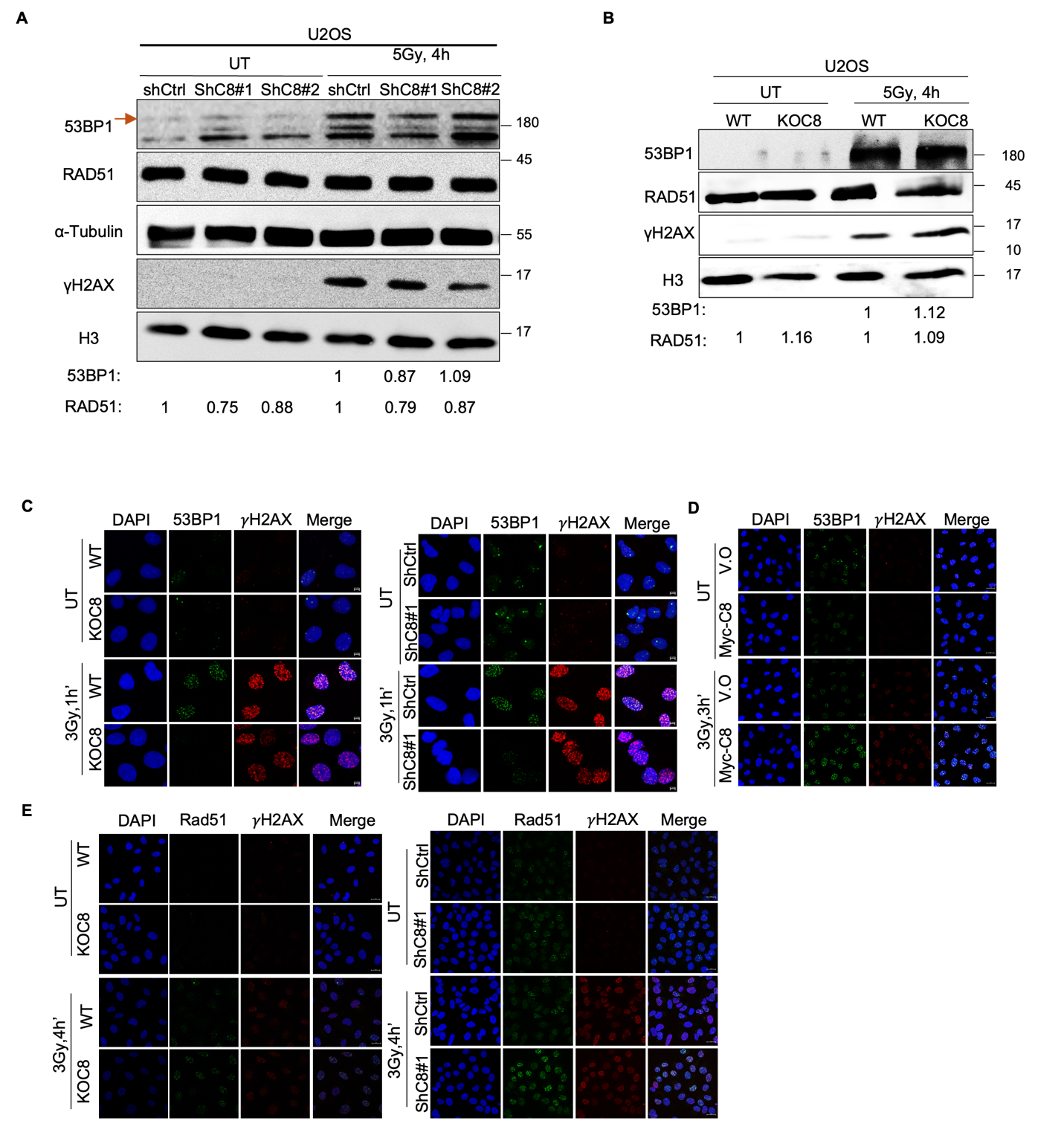
**

**Supplementary Figure 2:** Related to Figure 2-3.  **(A-B)** Western blot of U2OS cells before and after 5Gy IR induction to detect the global levels of 53BP1 and RAD51 upon C8orf33 depletion. (A) U2OS-ShC8#1, ShC8#2 and ShCtrl. (B). U2OS-WT and C8orf33 knockout (KOC8). Antibodies, 53BP1, RAD51, C8orf33, γH2AX, marker of DSB, H3 and tubulin were loading controls. The positions of molecular weight markers are indicated to the right. The orange arrow indicates 53BP1 protein. **(C)** Representative images of 53BP1 foci in U2OS-proficient and deficient cells before and after IR. Left, U2OS-WT and U2OS-KOC8. Right, U2OS-ShCtrl and U2OS-ShC8. Cells were co-stained for γH2AX (red) and 53BP1 (green) DNA was stained with DAPI (blue). Scale Bar 5 µm **(D)** Representative image of 53BP1 foci in U2OS-V.O and U2OS-Myc-C8 cells. Cells were co-stained for γH2AX (red) and 53BP1 (green) DNA was stained with DAPI (blue). Scale Bar 20 µm **(E)** Representative images of RAD51 foci in U2OS-proficient and deficient cells before and after IR. Left, U2OS-WT and U2OS-KOC8. Right, U2OS-ShCtrl and U2OS-ShC8. Cells were co-stained for γH2AX (red) and RAD51 (green) DNA is stained with DAPI (blue).


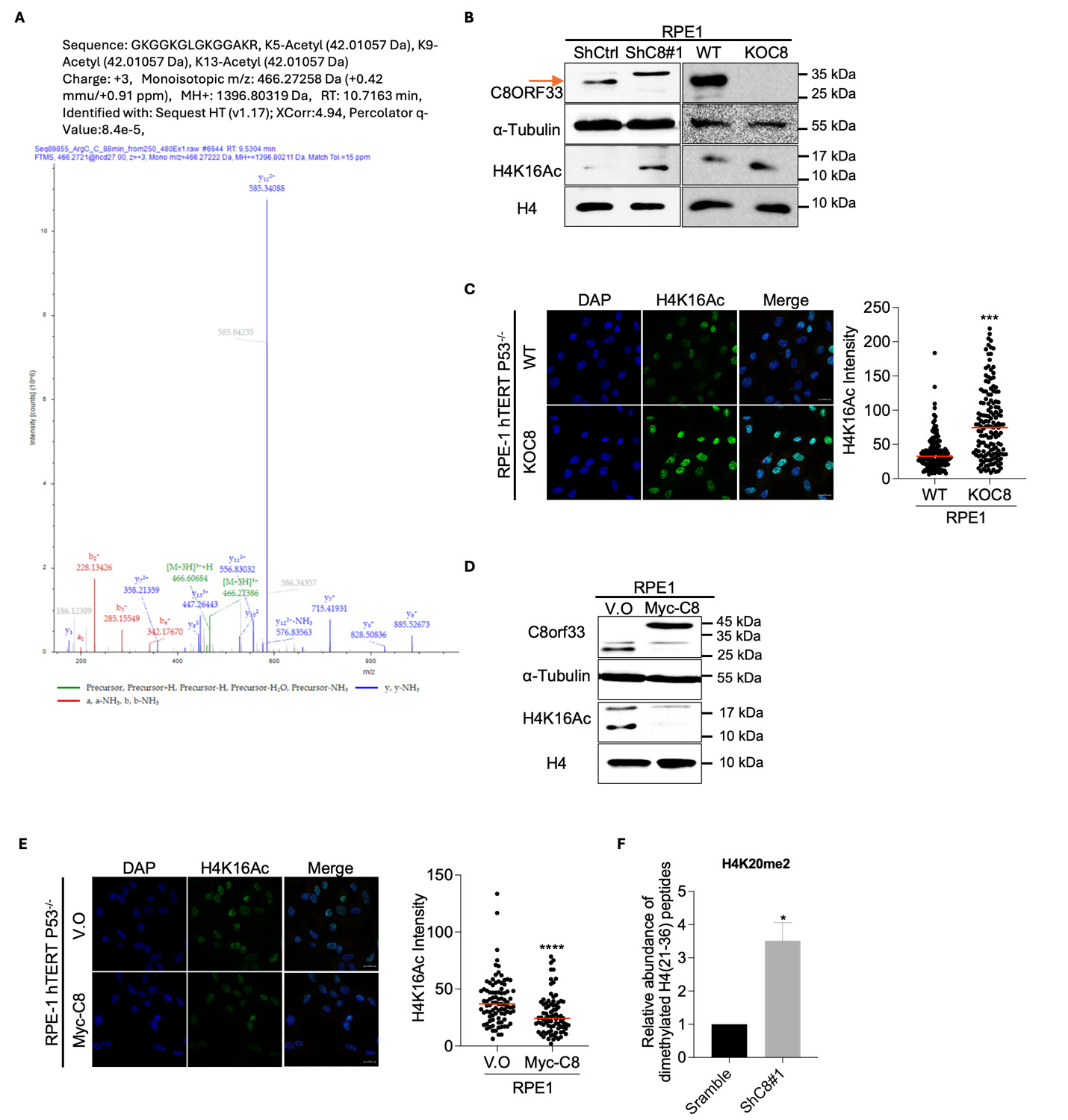


**Supplementary Figure 3:** Related to Figure 4. **(A)** Mass spectrometry spectrum of K8, K12 and K16 acetylated H4(5-18) peptide as identified by MS. **(B)** Western blot analysis in C8orf33 proficient and deficient RPE1 cells. Antibodies used, C8orf33, H4K16Ac, Tubulin, H4 antibodies. Tubulin and H4 were used as loading controls. The positions of molecular weight markers are indicated to the right. Orange arrow marks C8orf33 protein. **(C)** Mean intensity of H4K16ac in RPE1 proficient and deficient cells. Left, Representative images of H4K16Ac staining (green) and DNA staining with DAPI. Right quantification of H4K16Ac mean intensity per cell in RPE1 cells. Horizontal bars represent mean value of H4K16ac intensity per cell ± SEM for N>90. Scale Bar 20 µm **(D)** Western blot analysis of RPE1 cells expressing either V.O or Myc-C8 fusion vector. Antibodies used, C8orf33, H4K16Ac, Tubulin, H4 antibodies. Tubulin and H4 were used as loading controls. The positions of molecular weight markers are indicated to the right. **(E)** Mean intensity of H4K16ac in RPE1 overexpressing Myc-C8. Left, Representative images of H4K16Ac staining (green) and DNA staining with DAPI. Right quantification of H4K16Ac mean intensity per cell in RPE1 cells (left) Horizontal bars represent mean value of H4K16ac intensity per cell ± SEM for N>90. Scale Bar 20 µm *P* values were determined by two-tailed Mann-Whitney test. ns is not significant, ^*^p>0.05, ^∗∗^p < 0.01 and ^***^p>0.000. **(F)**. Results of quantitative mass spectrometry analysis of H4(28-42) di-methylated peptides in U2OS ShCtrl and ShC8. Error bars represent ±SD of N=2. modified H4 peptides’ intensity were normalized to the intensity of total peptides of H4. *P* value was determined by two-sided Students *t*-test relative to control cells. 1.

**
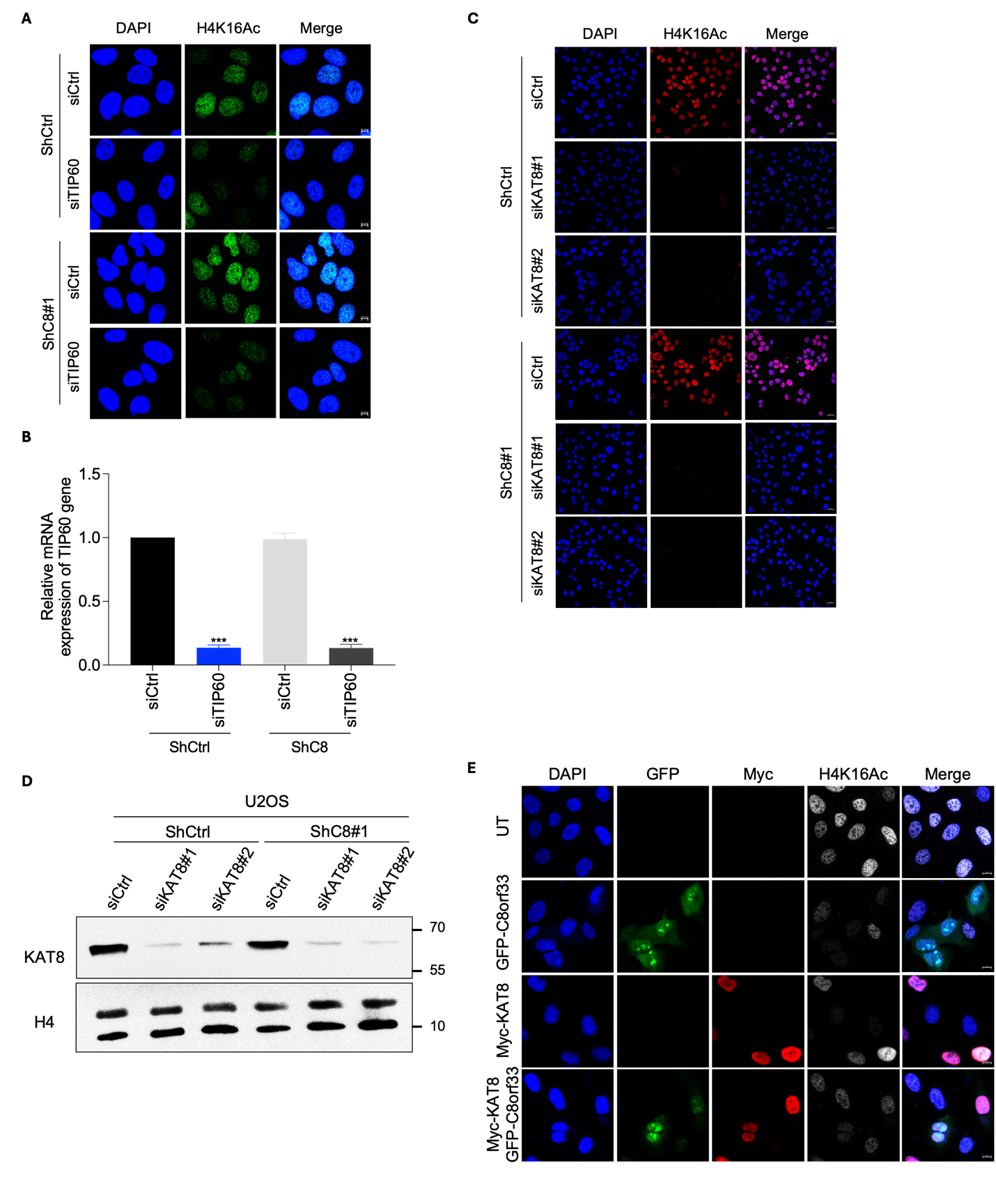
 Supplementary Figure 4:** Related to Figure 4. **(A)** Representative Image of mean intensity of H4K16ac in C8orf33 deficient and C8orf33 and TIP60 mutually deficient cells. (green) H4K16ac DNA was stained with DAPI. **(B)** Representative Image of mean intensity of H4K16ac in C8orf33 deficient and C8orf33 and KAT8 mutually deficient cells. (Red) H4K16ac DNA was stained with DAPI. **(C)** relative mRNA expression of TIP60 gene in ShCtrl and ShC8 cells. The mRNA was normalized to GAPDH gene. Error bars represent ±SD of N=3. *P* value was determined two-sided Students *t*-test relative to control cells. **(D)** Western blot analysis of U2OS cells transfected with either Ctrl siRNA or siRNA against KAT8 in ShCtrl and ShC8. antibodies used, KAT8 and H4 antibodies, H4 was used as loading control. **(E)** Representative images of mean intensity of H4K16ac. Un-transfected cells, pEGFP-C8, Myc-KAT8 and cell co-expressing GFP-C8orf33 and Myc-KAT8. Myc was stained with red, H4K16ac was stained with far-red, and DNA was stained with DAPI.


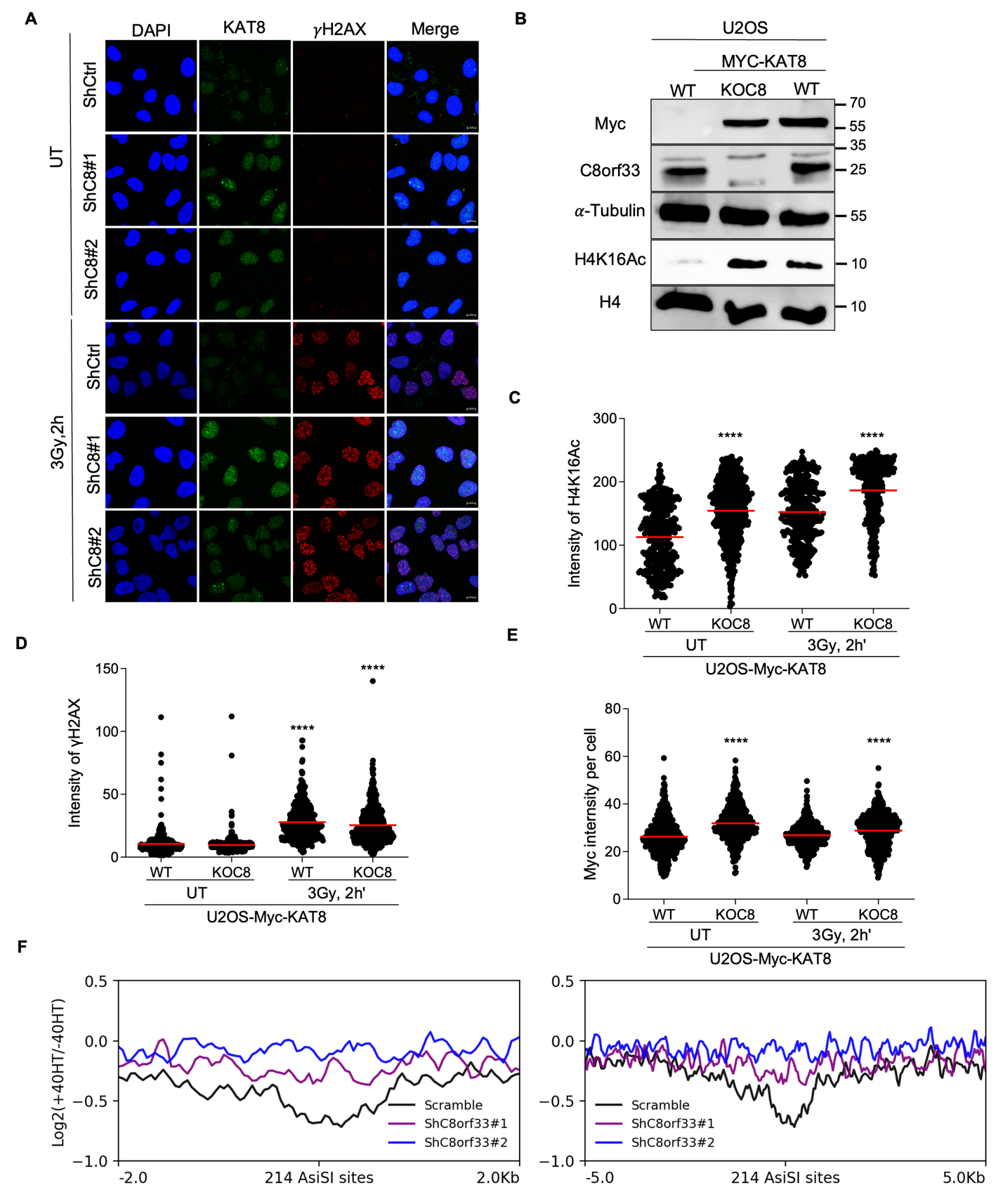


**Supplementary Figure 5:** Related to Figure 5. **(A)** Representative image of KAT8 chromatin association (green) co-stained with H4K16ac (farred) and γH2AX (Red). DNA was stained with DAPI. Scale bar 10 µm **(B)** Western blot for Myc-KAT8 expression in C8orf33 proficient and deficient U2OS cells. Antibodies used, Myc, C8orf33, tubulin, H4K16ac and H4. H4 and tubulin were used as loading controls. The positions of molecular weight markers are indicated to the right. **(C-E)** Quantification of mean intensity per cell for Myc (C), H4K16ac (D) and γH2AX (E). Horizontal bars represent mean value of intensity per cell ± SEM for N>300. *P* values were determined by two-tailed Mann-Whitney test. **(F)** Distribution of KAT8 log2 ratio between 4OHT-treated and 4OHT-untreated for DIvA cells (ShCtrl, ShC8#1 and ShC8#2) surrounding the 214 cleaved *Asi*SI sites. Left, distribution at 2 Kb window. Right, distribution at 5Kb window. The ChIP-seq data represents 3 independent biological repeats.

**
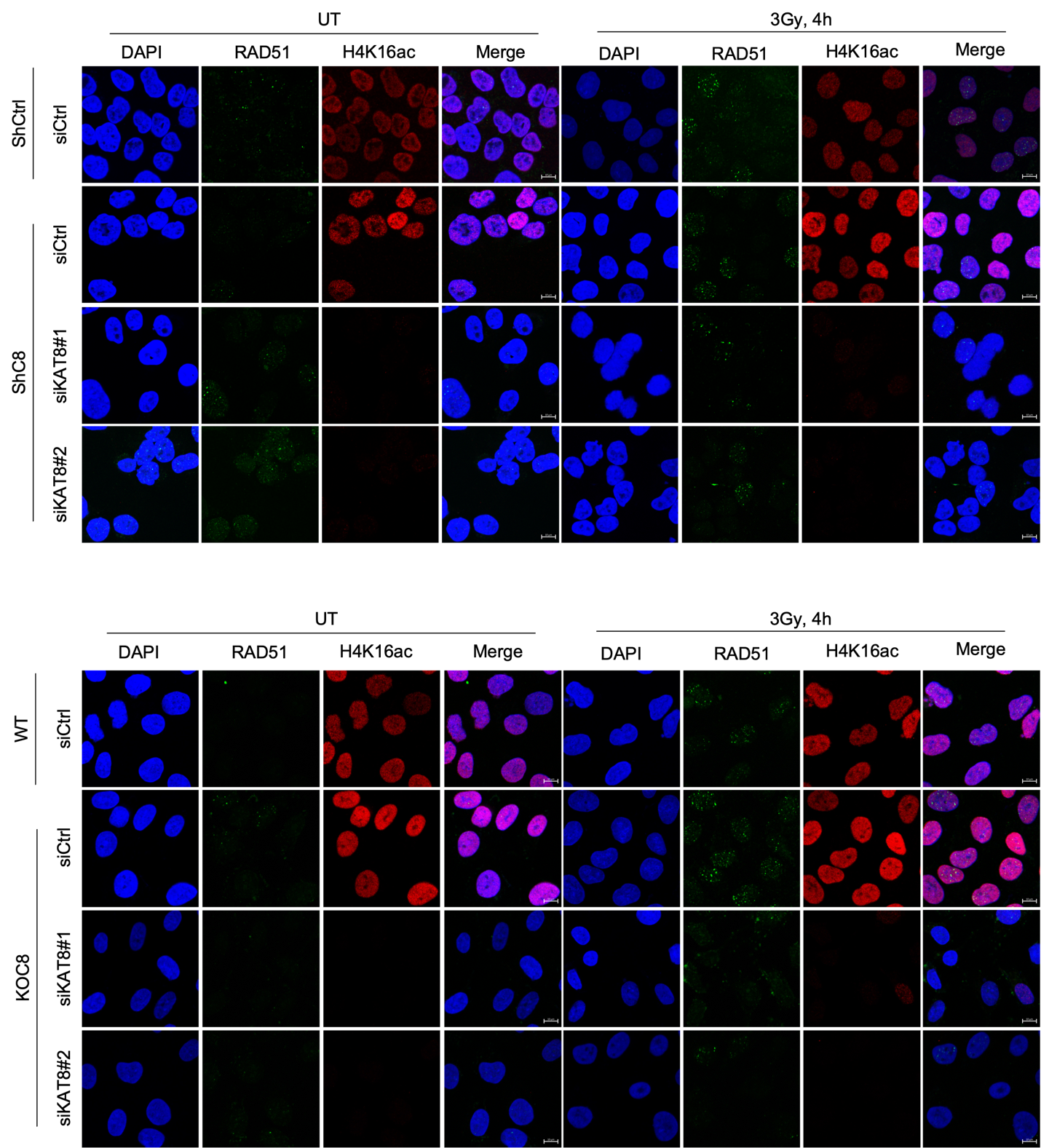
 Supplementary Figure 6:** Related to Figure 6. Representative image of H4K16ac intensity and RAD51 foci per cell both before and after IR induction (3Gy, 4h recovery) in U2OS cells either C8orf33 deficient or co-depleted of C8orf33 and KAT8. RAD51 (green) co-stained with H4K16ac (farred). DNA was stained with DAPI. Scale bar 10 µm

**
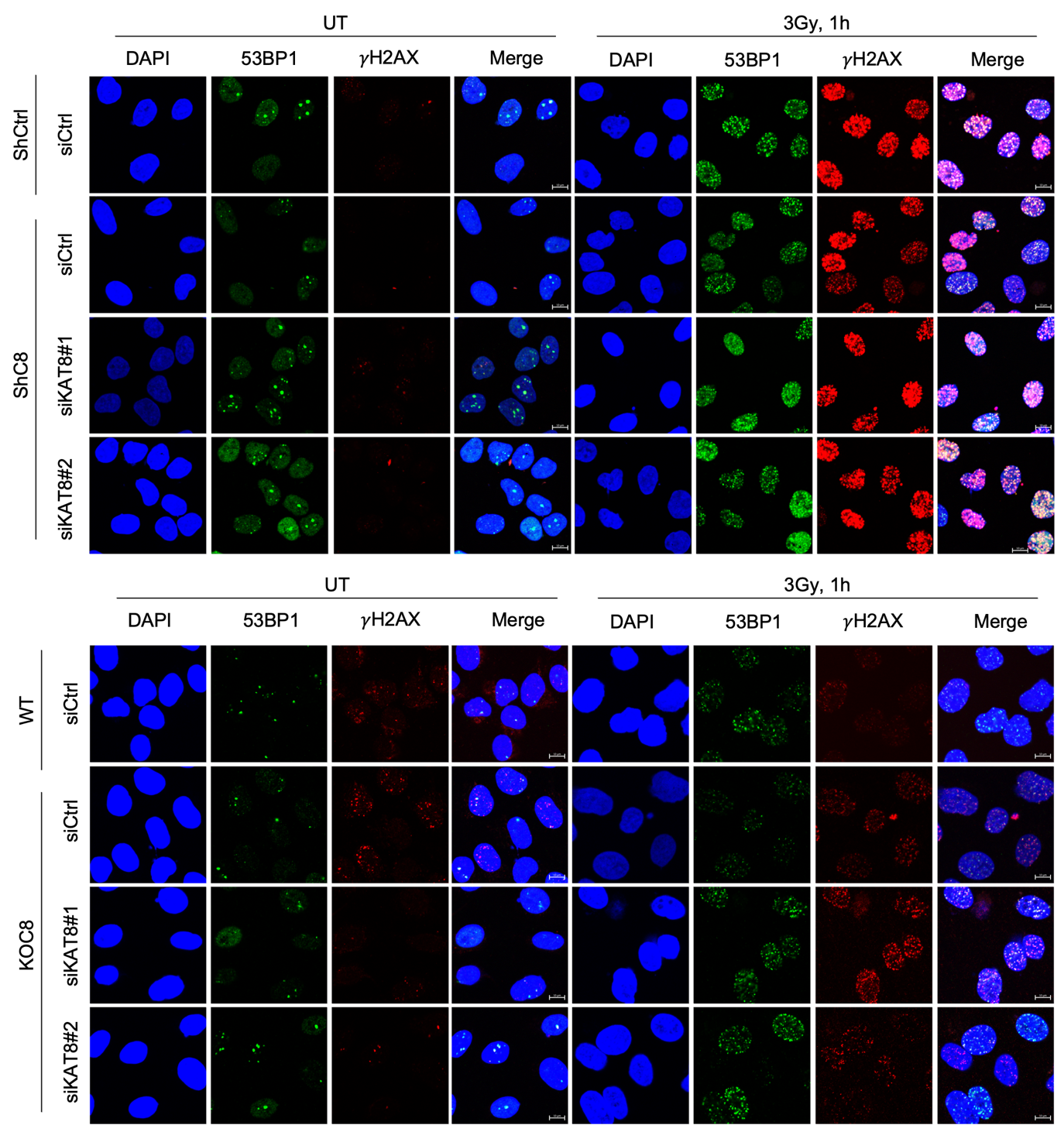
 Supplementary Figure 7:** Related to Figure 6. Representative image of γH2AX and 53BP1 foci per cell both before and after IR induction (3Gy, 4h recovery) in U2OS cells either C8orf33 deficient or co-depleted of C8orf33 and KAT8. 53BP1 (green) co-stained with γH2AX (red). DNA was stained with DAPI. Scale bar 10 µm.

**
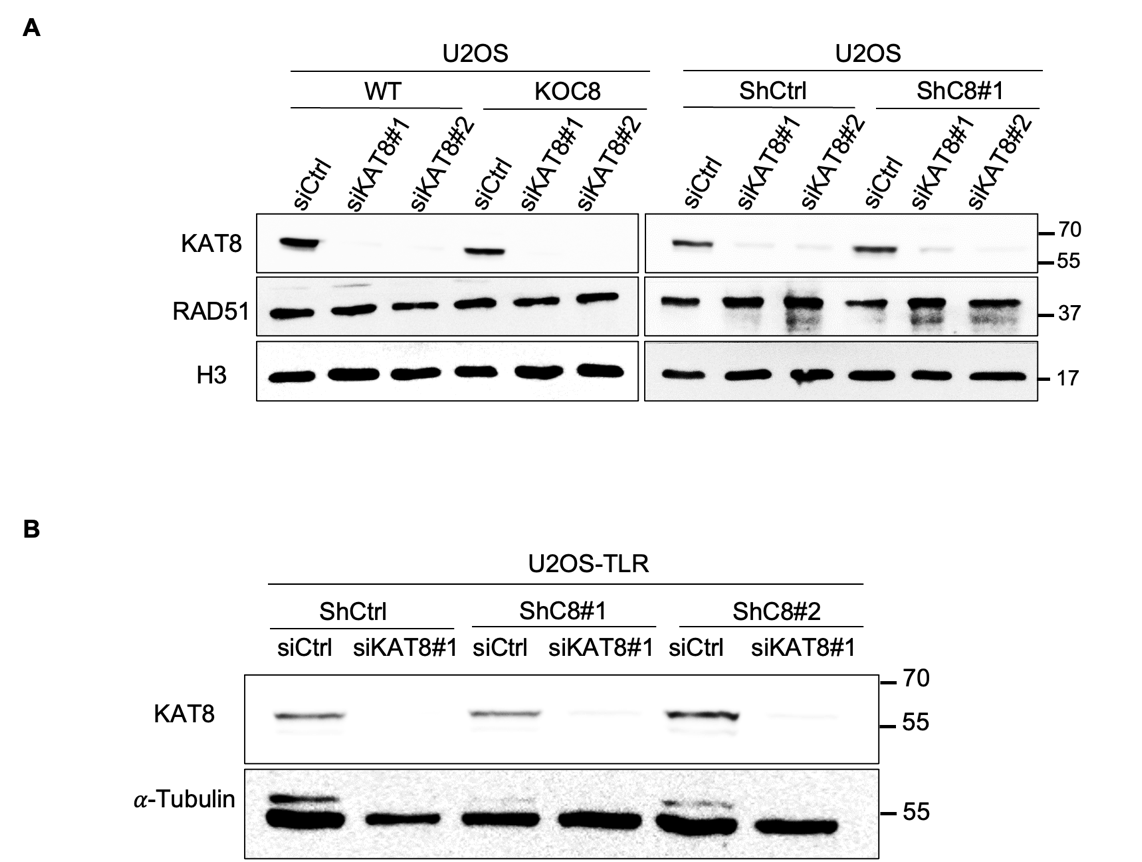
**

**Supplementary Figure 8:** Related to Figure 6. **(A)** Western blot analysis of U2OS cells transfected with either Ctrl siRNA or siRNA against KAT8. Left, WT and KOC8. Right, shCtrl and ShC8. antibodies used, KAT8, RAD51 and H3 antibodies, H3 was used as loading control. **(B)** Western blot for U2OS-TLR-ShCtrl or ShC8 transfected with siCtrl or siRNA for KAT8. antibodies, KAT8 and tubulin which is used as a loading control. The positions of molecular weight markers are indicated to the right.

**
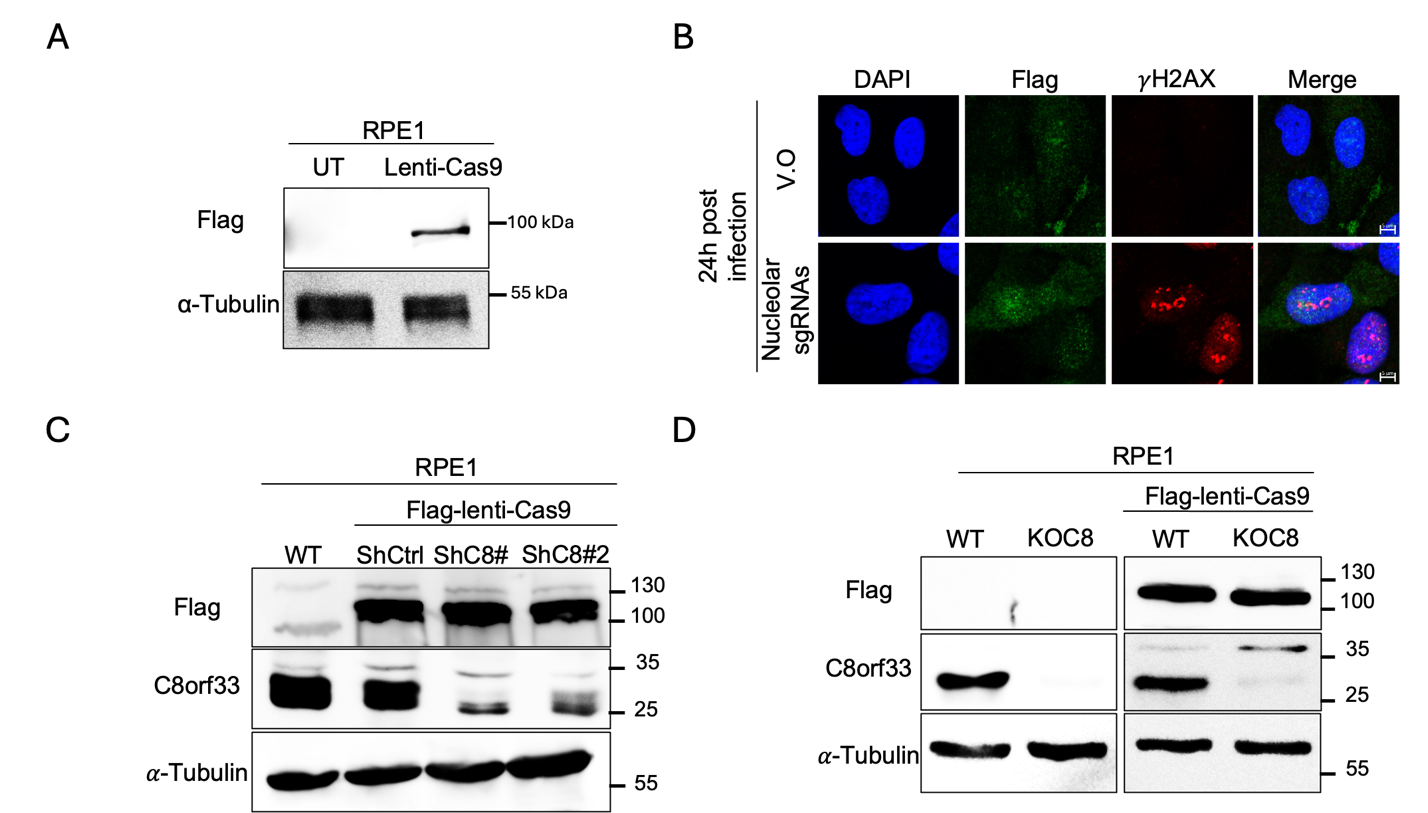
**

**Supplementary Figure 9:** Related to Figure 7. **(A)** Western blot showing the stable expression of pLV-Flag-Cas9 vector in RPE1 cells. Antibodies used, Flag and tubulin as loading control. The positions of molecular weight markers are indicated to the right. **(B)** Representative image of RPE1 stably expressing Flag-Cas9 infected with V.O or sgRNAs mix targeting the nucleolus. 24h post infection the cells were stained with flag (green) and γH2AX (red). Scale bar 5 µm. **(C-D)** Western blot analysis for Flag-Cas9 expression in RPE1 cells. (C) ShCtrl or ShC8. (D) WT and KOC8. Antibodies used Flag, C8orf33 or tubulin as loading control. **(D)** Western blot analysis for Flag-Cas9 expression in C8orf33 proficient and deficient RPE1 cells transfected with siCtrl or siKAT8. Antibodies used Flag and tubulin as loading control. The positions of molecular weight markers in all western blot are indicated to the right.

**Supplementary tables:**

| **Table S1: Reagents or resource source identifier** | | |
| --- | --- | --- |
| **Antibodies** | | |
| Rabbit anti-C8orf33 Antibody  (WB 1:5,000) | BETHYL | A305-873A |
| Rabbit anti-phospho-Histone H2A.X (Ser139)  (WB 1:1,000) | Cell-Signaling | 2577 |
| Rabbit anti-α-Tubulin (WB 1:2,000) | Cell-Signalling | CST-2125 |
| Rabbit polyclonal anti-Histone H3 (WB 1:30,000) | Abcam | ab1791 |
| c-Myc 9E10 (WB 1,1000, ChIP 1µg) | santa-cruz | SC-40 |
| Mouse monoclonal anti-phospho-Histone H2A.X  (Ser139), clone JBW301 (IF 1:1,2500) | Millipore | 05-636 |
| Rabbit anti-53BP1(WB 1:1,1000) | novus biological | NB100-305 |
| Rabbit anti-phospho RPA32 (S4/S8) (ChIP 1µg) | BETHYL | A300-245A |
| Rabbit anti-Rad51 (H-92) (ChIP 1µg) | santa cruz | SC-8349 |
| Mouse anti-Rad51(WB 1:1,1000, IF 1:2,000) | Abcam | ab88572 |
| Mouse anti-BRCA1 (D9) (ChIP 1µg) | santa cruz | SC-6954 |
| Rabbit H4K16ac (WB 1:1,000, IF 1:500, ChIP 1µg) | Abcam | ab109463 |
| Rabbit anti-Histone H4 (WB 1:3,000) | Abcam | ab177840 |
| Rabbit anti-KAT8 (WB 1:3,000, IF 1:2,000, ChIP 2µg) | Abcam | ab200660 |
| Rabbit anti-Flag (WB 1:3,500, IF 1:1,000) | sigma | F7425 |
| **Bacterial and virus strains** | | |
| DH5α Competent Cells | Thermo Scientific™ | EC0112 |
| ElectroMax Stbl4 | Thermo Scientific™ | Invitrogen™11635018 |
| **Chemicals and recombinant proteins** | | |
| Caffeine | Sigma-Aldrich | C8961 |
| ATMi | Sigma-Aldrich | KU-60019 |
| Ethidium bromide (EtBr) | Hylabs | BP451 |
| Polyethylenimine (PEI) | Polysciences | 343-6484 |
| Hexadimethrine bromide (Polybrene) | Sigma-Aldrich | H9268 |
| Puromycin  (z)-4-Hydroxytamoxifen | Invivogen  Sigma-Aldrich | ant-pr H7904 |
| BLASTICIDIN | Invivogen | ANT-BL-1 |
| Hygromycin B | MERCK | 400052 |
| G418 | CALBIOCHEM | 345810 |
| SeaPlaque® Agarose | LONZA | 50101 |
| **Critical commercial assays** | | |
| WesternBright Quantom (ECL) | Advansta | K-12042 |
| Fast SYBR green master mix | Applied Biosystems | 4385610 |
| Phusion® High-Fidelity DNA Polymerase | NEB | M0530 |
| Protein A Magnetic bead | Invitrogen | 10002D |
| Protein G magnetic beads | Gene Script | L00274 |
| PFA | Electron Microscopy Science | 15710 |
| MACHEREY-NEGEL Nucleospin kit | Ornat | 740609 |
| Exgene Cell SV mini | GeneAll | 10623H28069 |
| GraphPad Prism (v10.0) | GraphPad  software lnc. | https://www.graphpad.com |
| ImageJ software (v1.8.0) | National Institutes of Health | https://imagej.nih.gov/ij/ |

| **Table S2: Plasmids** | | |
| --- | --- | --- |
| pEGFP-C1 | Clontech Laboratories | #6084 |
| pDsRED-monomer-C1 | Clontech Laboratories | 632466 |
| pLKO.1 - TRC cloning vector | Addgene | #10878 |
| pMD2.G | Addgene | #12259 |
| psPAX2 | Addgene | #12260 |
| pX330-LMNA-gRNA1 | (1) | N/A |
| pCR2.1-CloverLamin | (1) | N/A |
| pX330-U6-Chimeric_BB-CBh-hSpCas9 | Addgene | #42230 |
| pLCKO | Addgene | #73311 |
| Lenti‐Cas9‐2A‐Blast | Addgene | #73310 |
| Psin-3xMyc-KAT8 | (2) | N/A |

| **Table S3: Plasmids generated in this study** | | |
| --- | --- | --- |
| **Plasmid** | **Vector backbone** | **Insert** |
| pEGFP-C8orf33-C1 | pEGFP-C1 | PCR product from cDNA using F1, R1 primers |
| PLKO.1-TRC-Scramble | pLKO.1 - TRC cloning vector digested with EcoRI, AgeI | Annealed primers F4, R4 |
| PLKO.1-TRC-C8orf33-shRNA1 | pLKO.1 - TRC cloning vector digested with EcoRI, AgeI | Annealed primers F2, R2 |
| PLKO.1-TRC-C8orf33-shRNA2 | pLKO.1 - TRC cloning vector digested with EcoRI, AgeI | Annealed primers F3, R3 |
| pX330-C8orf33-gRNA | pX330-U6-Chimeric_BB-CBh-hSpCas9 digested with BbsI | Annealed primers F5, R5 |
| pLV-C8orf33-T2A-Blast | pLV-RBM6-T2A-Blast (3) digested BamHI, ECORV | PCR product from pEGFP-C1-C8orf33 using primers F6, R6 |
| pLCKO-nuc-gRNA1 | pLCKO cloning vector digested with BfuAI | Annealed primers F7, R7 |
| pLCKO-nuc-gRNA2 | pLCKO cloning vector digested with BfuAI | Annealed primers F8, R8 |
| pLCKO-nuc-gRNA3 | pLCKO cloning vector digested with BfuAI | Annealed primers F8, R8 |
| pLCKO-nuc-gRNA4 | pLCKO cloning vector digested with BfuAI | Annealed primers F8, R8 |

| **Table S4: cloning primers** | |
| --- | --- |
| **Primer** | **Sequence** |
| F1 | ATAGTCGACATGGCGGCCCTGGGACATCTTGCTGGGGA |
| R1 | GCGGGGCCCCTAAAAGAAATTGAACCTAAACTCTTCATC |
| F2 | CCGGGATGAAGAGTTTAGGTTCAATCTCGAGATTGAACCTAAACTCTTCATCTTTTTG |
| R2 | AATTCAAAAAGATGAAGAGTTTAGGTTCAATCTCGAGATTGAACCTAAACTCTTCATC |
| F3 | CCGGCACTCCTTGTTTGGAGACTATCTCGAGATAGTCTCCAAACAAGGAGTGTTTTTG |
| R3 | AATTCAAAAACACTCCTTGTTTGGAGACTATCTCGAGATAGTCTCCAAACAAGGAGTG |
| F4 | CCGGGTGGACTCTTGAAAGTACTATCTCGAGATTTGACGGGTGGATAATCTGTTTTT |
| R4 | AATTAAAAACAGATTATCCACCCGTCAAATCTCGAGATAGTACTTTCAAGAGTCCAC |
| F5 | CACCGTGCCGCCTTCATCGCCCAA |
| R5 | AAACTTGGGCGATGAAGGCGGCAC |
| F6 | ACCGGCGAGAACAGCAGGCCCGC |
| R6 | AAACGCGGGCCTGCTGTTCTCGC |
| F7 | ACCGGCCTTCTCTAGCGATCTGAG |
| R7 | AAACCTCAGATCGCTAGAGAAGGC |
| F8 | ACCGGATTTCCAGGGACGGCGCCT |
| R8 | AAACAGGCGCCGTCCCTGGAAATC |
| F9 | ACCGGCGGTGCGTGACGGGCGAGG |
| R9 | AAACCCTCGCCCGTCACGCACCGC |
| **ChIP primers** | |
| F1 (DSB-I) | GTGTCAGGCGTTCTCGTCTC |
| R1 (DSB-I) | AGCACGACGTCACCACATC |
| F2 (DSB-II) | ACTCAGGGAACTCCATTGGC |
| R2 (DSB-II) | TACATCCGATTCGAGCCCTG |
| F3 (DSB-III) | GGGCTTCACCATCTTTCTGACA |
| R3 (DSB-III) | TGCTGGGGTCCCTCCTCCAAGT |
| **rDNA repeats detection primer** | |
| F (18S) | ACCACATCCAAGGAAGGCAG |
| R (18S) | CGCTATTGGAGCTGGAATTAC |
| TIP60-RT-qPCR primers | |
| F (TIP60) | CAACCACCGCTCAACGAAAC |
| R (TIP60) | AGAAGTACCACGGCTTGAGG |
| F(GAPDH) | CCAGGGCTGCTTTTAACTCT |
| R(GAPDH) | GGTGCCATGGAATTTGCCAT |
| **End resection primers** | |
| **Taqman- for DSB-1 and no DSB** | |
| F1-364 bp | CCAGCAGTAAAGGGGAGACAGA |
| R1-364 bp | CTGTTCAATCGTCTGCCCTTC |
| Probe | 6FAM-CCAGGCCCTCAAAATCCCTCCACTG-TAMRA |
| F2-1754 bp | GAAGCCATCCTACTCTTCTCACCT |
| R2-1754 bp | GCTGGAGATGATGAAGCCCA |
| Probe | 6FAM-CACTCCCTGTTCTTCTTCTGCTCCCGA-TAMRA |
| F3-3564 bp | GCCCAGCTAAGATCTTCCTTCA |
| R3-3564 bp | CTCCTTTGCCCTGAGAAGTGA |
| Probe | 6FAM-CTGCAGCCCTCAAGCCCGGAT-TAMRA |
| F4-noDSB | ATTGGGTATCTGCGTCTAGTGAGG |
| R4-noDSB | GACTCAATTACATCCCTGCAGCT |
| Probe | 6FAM-TCTCTGCACAGACCGGCTTCCCTTC-TAMRA |
| **Syber – for DSB-2** | |
| F5-335 bp | TGAGGAGGTGACATTAGAACTCAGA |
| R5-335 bp | AGGACTCACTTACACGGCCTTT |

**Supplementary references**:

1. Pinder J, Salsman J, Dellaire G. Nuclear domain 'knock-in' screen for the evaluation and identification of small molecule enhancers of CRISPR-based genome editing. Nucleic Acids Res. 2015;43(19):9379-92.

2. Wu Y, Zhou L, Zou Y, Zhang Y, Zhang M, Xu L, et al. Disrupting the phase separation of KAT8-IRF1 diminishes PD-L1 expression and promotes antitumor immunity. Nat Cancer. 2023;4(3):382-400.

3. Machour FE, Abu-Zhayia ER, Awwad SW, Bidany-Mizrahi T, Meinke S, Bishara LA, et al. RBM6 splicing factor promotes homologous recombination repair of double-strand breaks and modulates sensitivity to chemotherapeutic drugs. Nucleic Acids Res. 2021;49(20):11708-27.
