# Supplementary material for: C8orf33 dictates DNA double-strand break repair choice by modulating KAT8-mediated H4K16 acetylation
Source: Cell Death Dis. 2025 Nov 17;16(1):834. doi: 10.1038/s41419-025-08194-8 (PMC12624059; doi:10.1038/s41419-025-08194-8)
Supplement: Supplementary file 2 — Original western blots [file 41419_2025_8194_MOESM2_ESM.docx]

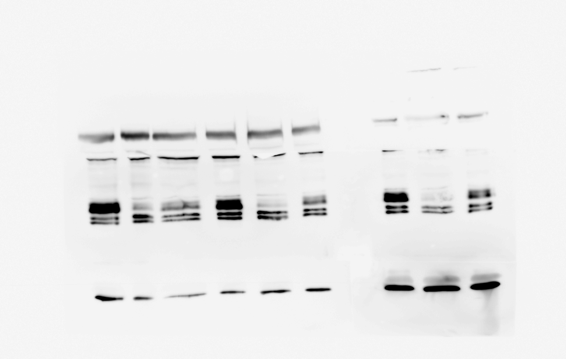

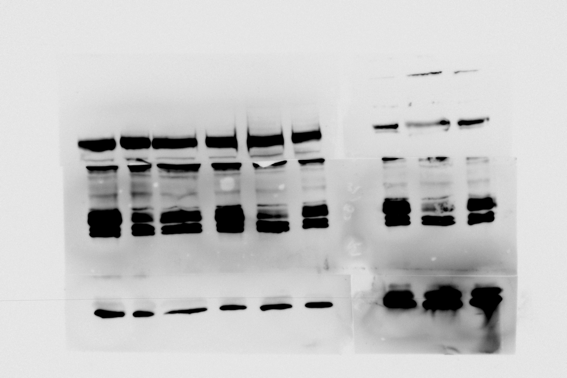

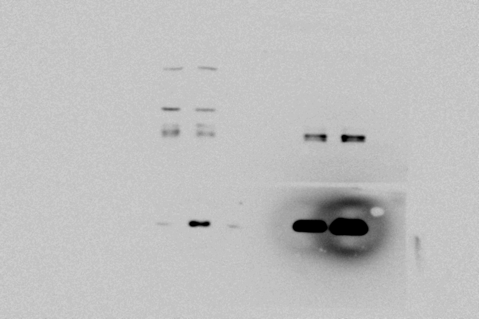

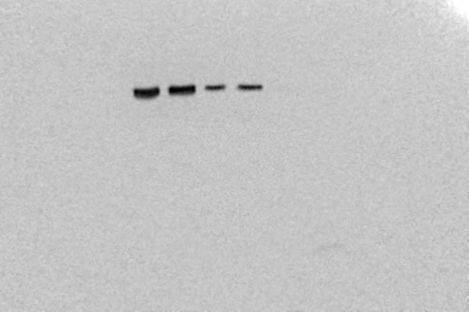

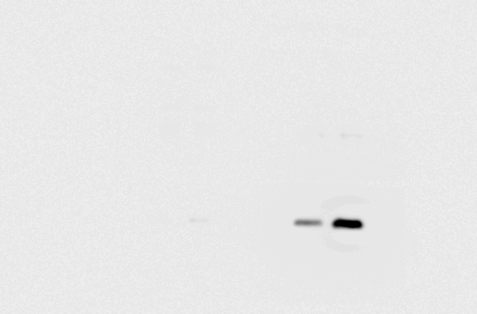

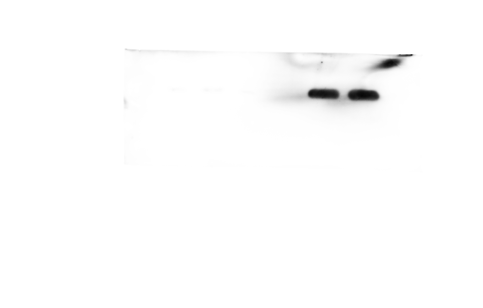

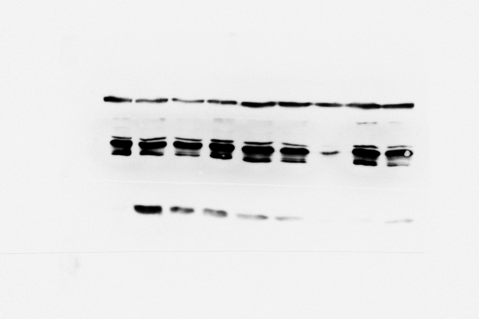

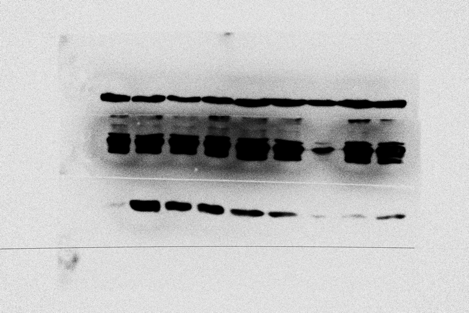

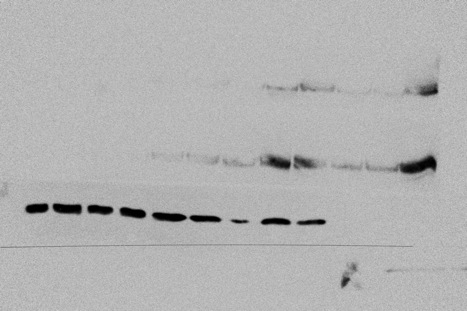

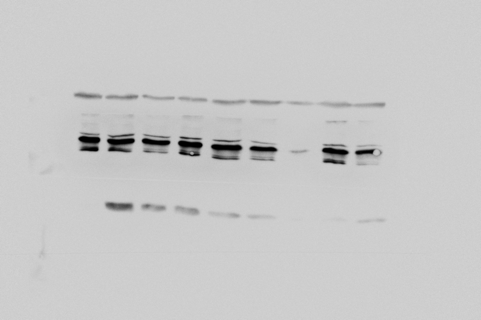

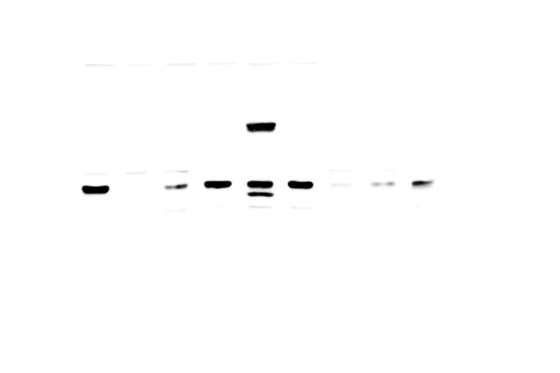

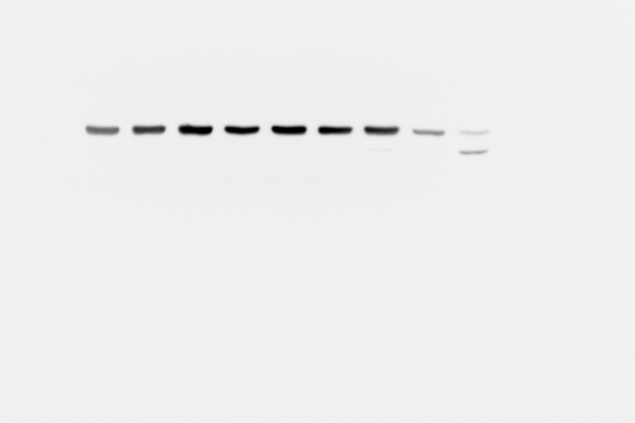

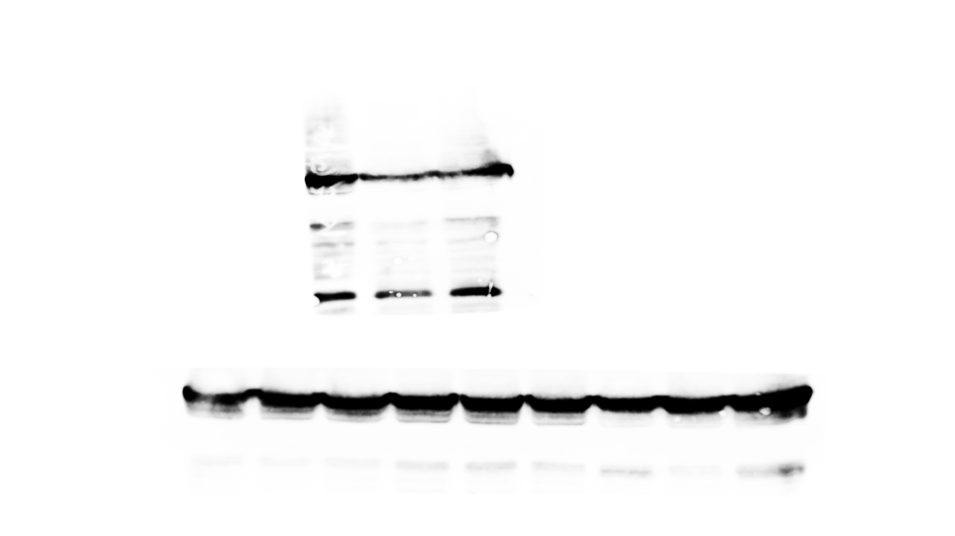




Figure 1K

Figure 1A

Figure 1B

Figure 1J




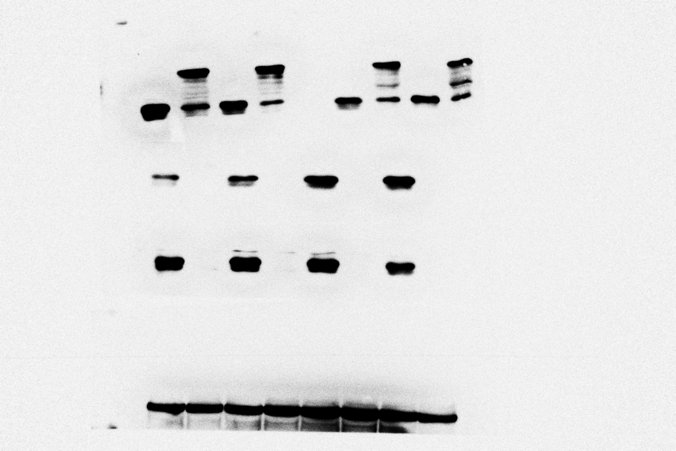

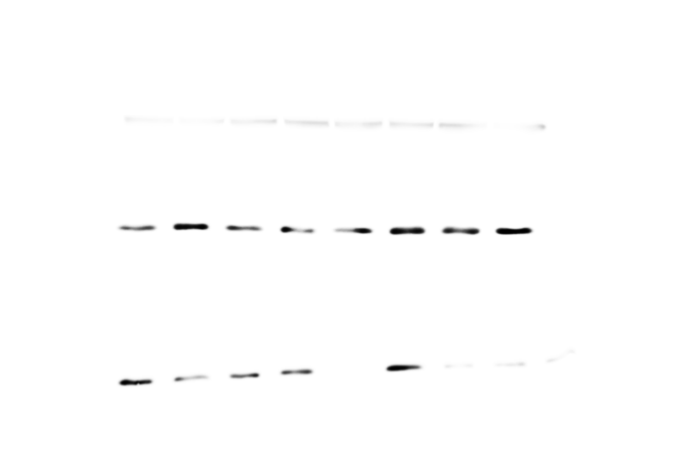

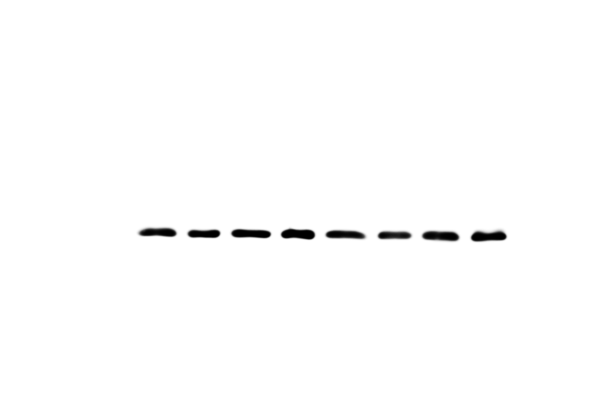














Figure 4A -H4K12ac

Figure 4A -H4K16ac




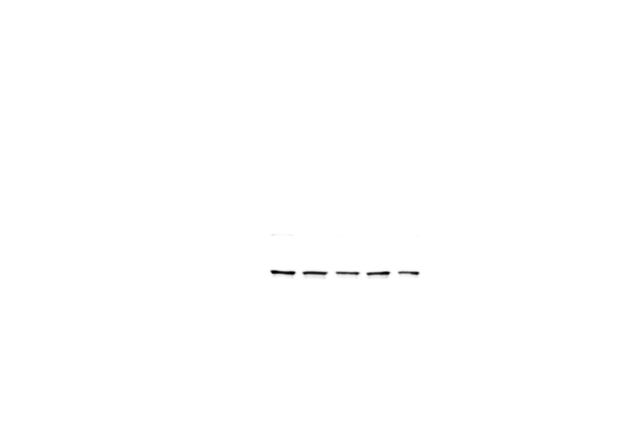




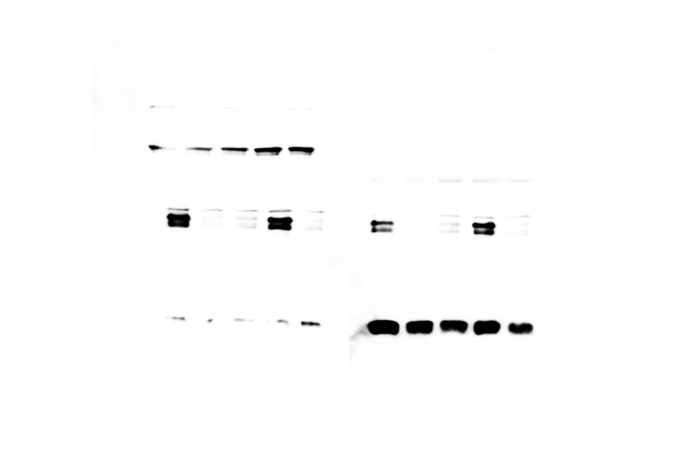

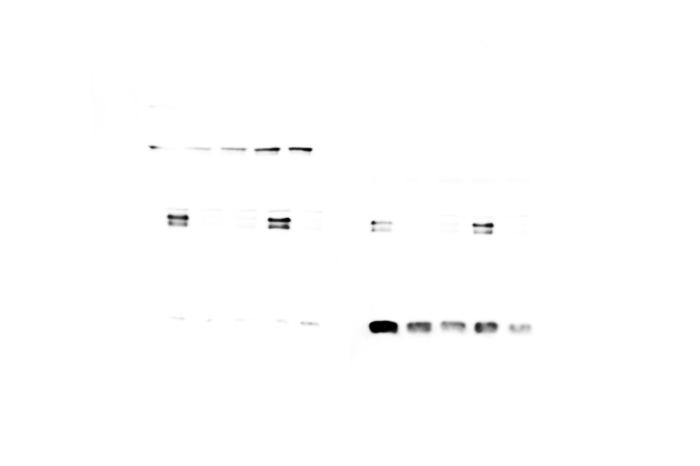


Figure 5A





Figure 4D







Figure S1C

Figure S1B































Figure S2B

Figure S2A
















Figure S3D

Figure S3B

























Figure S4D

Figure S5B









Figure S6A

Figure S6B




































Figure S8D

Figure S8C

Figure S8A
